# Single Atom Cocatalysts in Photocatalysis

**DOI:** 10.1002/adma.202414889

**Published:** 2024-12-29

**Authors:** Si‐Ming Wu, Patrik Schmuki

**Affiliations:** ^1^ Department of Materials Science WW4‐LKO University of Erlangen‐Nuremberg Martensstraße 7 91058 Erlangen Germany; ^2^ Regional Centre of Advanced Technologies and Materials Šlechtitelů 27 Olomouc 78371 Czech Republic

**Keywords:** cocatalyst, hydrogen production, photocatalysis, single atom

## Abstract

Single‐atom (SA) cocatalysts (SACs) have garnered significant attention in photocatalysis due to their unique electronic properties and high atom utilization efficiency. This review provides an overview of the concept and principles of SA cocatalyst in photocatalysis, emphasizing the intrinsic differences to SAs used in classic chemical catalysis. Key factors that influence the efficiency of SAs in photocatalytic reactions, particularly in photocatalytic hydrogen (H_2_) production, are highlighted. This review further covers synthesis methods, stabilization strategies, and characterization techniques for common SAs used in photocatalysis. Notably, “reactive deposition” method, which often shows a self‐homing effect and thus achieves a maximum utilization efficiency of SA cocatalysts, is emphasized. Furthermore, the applications of SA cocatalysts in various photocatalytic processes, including H_2_ evolution, carbon dioxide reduction, nitrogen fixation, and organic synthesis, are comprehensively reviewed, along with insights into common artifacts in these applications. This review concludes by addressing the challenges faced by SACs in photocatalysis and offering perspectives on future developments, with the aim of informing and advancing research on SAs for photocatalytic energy conversion.

## Graphical abstract

Single‐atom cocatalysts (SACs) in photocatalysis are comprehensively discussed, with a focus on often‐overlooked aspects of general concepts and understanding. Key principles, challenges, and recent advancements are covered, highlighting the self‐homing effect as a promising avenue for designing SACs with maximized efficiency and unexpected features.

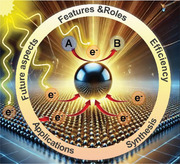

## 1 Introduction

Single‐atom catalysts (SACs) represent a significant advancement in the field of catalysis, pushing the boundaries of efficiency and selectivity down to the atomic level.^[^ ^1^, ^2^, ^3^ ^]^ These catalysts are—in the ideal case—individual metal atoms precisely positioned on suitable supports such as classic carbon‐based substrates,^[^ ^4^ ^]^ various metal oxides,^[^ ^5^ ^]^ or highly designed hosts such as metal–organic frameworks (MOFs) or zeolites.^[^ ^6^, ^7^ ^]^ In the ideal case SAs are fully accessible and therefore can provide full utilization, with each atom participating directly in the catalytic process. This maximizes the efficiency of the loaded active catalyst and allows to minimize the loading required to achieve a desired reaction rate. Scientifically even more spectacular are the unique electronic properties of SAs that in comparison to classic bulk or nanoparticles can result in enhanced and/or modified catalytic properties. That is, by using SAs, not only an enhancement of the specific reactivity can be reached but also alterations of the selectivity of a reaction.

**Figure 1:**
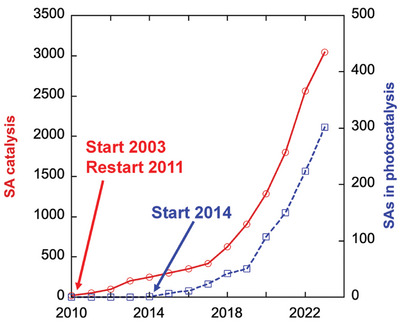
Number of publications on SA catalysis and SA photocatalysis from Web of Science.

Historically, the first reports of SA catalysis can be dated back to the remarkable work of Flytzani‐Stephanopoulos and co‐workers in 2003,^[^ ^8^ ^]^ they reported on the high reactivity of cationic single‐atom Au and Pt species supported on CeO_2_ for the water‐gas shift reaction. Later, in 2011, Qiao et al.^[^ ^9^ ^]^ described the use of Pt SAs on iron oxide (FeO*_x_*) for CO oxidation—this report additionally stimulated the field and contributed to triggering an almost exponential increase in publication rate on SA catalysis in the following years (**Figure** **1**). Meanwhile, SACs have been employed in catalyzing a wide array of classic chemical reactions, including hydrogenation, oxidation, water‐gas shift, dehydrogenation and reforming reactions, and hydroformylation, etc. The status of the field has been reviewed excessively in many excellent overviews.^[^ ^2^, ^3^, ^10^, ^11^, ^12^, ^13^ ^]^

More recently, SACs have found strong interest also in photocatalysis, namely, as so‐called cocatalysts. In photocatalysis, light illumination of a semiconductor generates charge carriers (e^−^–h^+^) in the semiconductor, the charge carriers then can migrate to the surface and react with the surrounding environment. However, many semiconductor surfaces provide only slow electron transfer kinetics to the target red/ox species; this is particularly impacting some important electrochemical reactions, such as hydrogen evolution reaction (HER), oxygen evolution reaction (OER), or CO_2_ reduction. Here suitable cocatalysts, decorated on the semiconductor surface, offer often a drastic kinetic increase of the reaction rate, and if these cocatalysts are used as SAs, an outstanding utilization efficiency can be reached (which is particularly interesting if the cocatalysts are noble metals). Moreover, in some cases surprising reactivities and alternative reaction pathways are enabled.^[^ ^14^, ^15^, ^16^, ^17^ ^]^ It is important to note that many critical features of SAs in photocatalysis, such as rate determining factors, role of support, or aggregation stability, are very different from the classic role of SAs in chemical catalysis—these specific differences will be a main part of this review.

The first report on the use of SAs in photocatalysis is by Xing et al.^[^ ^18^ ^]^ in 2014 who described single‐atom Pt and other noble metals anchored on anatase TiO_2_ for cocatalyzing the photocatalytic H_2_ evolution reaction. It was shown that several SA cocatalysts exhibited higher activity toward H_2_ evolution as compared to nanoparticle (NP) counterparts. The work by Xing et al.^[^ ^18^ ^]^ is remarkably comprehensive and considers a whole range of key aspects in the use of noble metal SAs for photocatalytic H_2_ generation.

In comparison with research work on SAs in classic chemical catalysis, the use of SAs in photocatalysis found wide attention with a delay of 4–5 years, and paper‐output entered an exponential phase only around 2019–2020 (Figure 1). At the time this review is written, SAs in both fields show a nearly exponential increase of publications per year.

In photocatalysis, SAs play meanwhile a pivotal role in driving the efficiency of key photocatalytic processes such as water splitting,^[^ ^19^ ^]^ CO_2_ reduction,^[^ ^20^ ^]^ waste degradation,^[^ ^21^ ^]^ as well as organic transformation reactions (such as in upconversion of pollutants to value‐added commodities).^[^ ^22^ ^]^ At the same time, constantly new work on fundamental aspects, addressing synthesis, anchoring, and use of SAs is released and currently represents a vivid research frontier.

**Figure 2:**
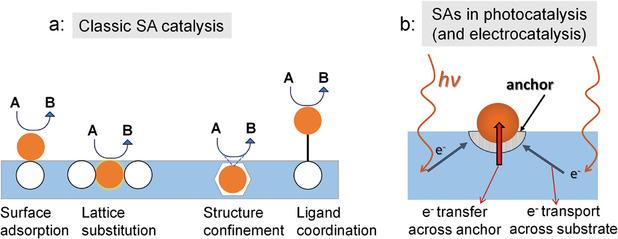
Schematic illustration of the difference between a) classic SA catalysis and b) SAs in photocatalysis (and electrocatalysis).

A main reason for us to write up a review on SAs in photocatalysis is not only that in contrast to SAs in classic catalysis, the specific role of SAs as cocatalysts has been much less addressed in review type of articles,^[^ ^22^, ^23^ ^]^ but even more the fact that many previous review articles are written from the view‐angle of classic catalysis while critical aspects of photocatalysts are not considered to an extent they deserve. Therefore, in the present review we emphasize crucial elements that need attention when dealing with SAs in photocatalysis (as well as in photoelectrochemistry), such as the rate controlling factors (low SA loading may be good enough), the nature of optical and electronic properties of the semiconductor, or the need of electrically well connected SAs anchored at the “right” surface sites (**Figure** **2**).

### 1.1 Differences in Use of SA in Photocatalysis versus Classic Catalysis

Major differences in use of SAs in photocatalysis to classic catalysis are the role of the substrate and the often‐different overall rate‐determining factor. In classic chemical SA catalysis, the substrate is commonly selected according to an optimized metal–substrate interaction,^[^ ^24^ ^]^ as outlined, e.g., by the Tauster‐principles.^[^ ^25^ ^]^ For example, the main electronic interaction between the substrate and the SA is of a static nature and it can (only) be altered by changing the substrate. In this case, a range of different anchoring types of SAs are possible (such as surface adsorbed, substitutionally embedded in a surface, fully buried in a substrate, or ligand coordinated), as illustrated in Figure 2a.

The catalytic atoms are (in principle) self‐sufficient to catalyze the reaction from the reactant (A) to the product (B). This may involve some different level of steric or diffusional hindrance and each anchoring site provides a static level of specific support interaction. However, in photocatalysis, electrocatalysis, or photoelectrocatalysis, the substrate plays a much more important role.

More specifically, in photocatalysis, the substrate is a photon absorber, in general a suitable semiconductor that is illuminated with super bandgap (solar) light. This means that access of light to the surface is needed, and the generated charge carriers not only need to reach the reactive site, but they also need to be transferred from support to the SA (Figure 2b). In other words, not only the electronic properties of the substrate (conductivity, electron mobility) play a key role but also electron transfer to the SA in order to enable a red‐ox reaction—the electronic anchoring of the SA in the substrate (the electron transfer across the interface substrate‐to‐SA) can drastically affect the reaction rate (this will be later discussed in some more detail).

In photocatalysis, often the availability of charge carriers is rate determining—rather than the reaction rate at the SA or the surface density of SAs. In other words, in contrast to classic chemical catalytic reactions, under common illumination conditions, not a high loading of SAs is needed but often a low density (10^5^–10^6^ SAs per µm^2^) is sufficient to obtain already a maximized cocatalytic efficiency.

Another point to consider is that in contrast to classic chemical catalysis or classic electrochemical reactions, in photocatalysis, electron transfer is inevitably coupled with hole transfer, where electron and hole transfer take place on the same surface (of, e.g., a suspended TiO_2_ particle). This is, a cocatalyst may be required to facilitate electron or hole transfer, whatever transfer reaction is rate‐determining.

Whether electron or hole transfer dictate the overall reaction rate, largely depends on the type of respective reactions at the conduction or valence band. Typical conduction band reactions are the reduction of water to dihydrogen (H_2_), the reduction of oxygen (O_2_) to O*_2_, the reduction of N_2_ to NH*_x_* compounds, or CO_2_ to hydrocarbon species, while typical valence band reactions are the oxidation of H_2_O to O_2_ (OER), the oxidative formation of radicals from water, or the oxidation of organics (e.g., many organic hydroxy compounds, alcohols, are kinetically much easier to be oxidized than water; therefore they are also frequently used as so‐called “sacrificial agents”). In general, many metallic species (such as Pt, Pd, Au, etc.) cocatalyze electron transfer reactions at the conduction band, whereas oxides (RuO_2_, IrO_2_) or hydroxide‐species catalyze hole transfer at the valence band.^[^ ^26^, ^27^ ^]^

## 2 Fundamentals of Photocatalysis and SAs as Cocatalyst

### 2.1 Photocatalysis and Cocatalysts

**Figure 3:**
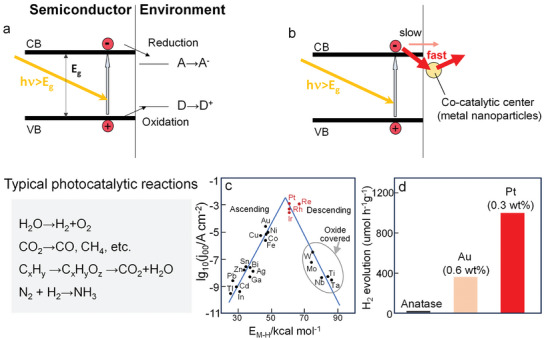
a) Schematic illustration of the mechanism of photocatalysis. b) Schematic illustration of the function of cocatalyst, and c) volcano plot for HER in acidic solutions. Reproduced with permission.^[^ ^47^ ^]^ Copyright 1972, Elsevier. d) Comparison of photocatalytic H_2_ production activity of anatase TiO_2_ and anatase TiO_2_ decorated with Au or Pt nanoparticles.

Photocatalytic reactions are based on the interaction of light with a semiconductor (**Figure** **3**a). Under super‐bandgap illumination, electron–hole pairs are created and these photogenerated charge carriers can migrate (or diffuse) on conduction and valence band, to the semiconductor surface and there react with suitable red‐ox species in the electrolyte. That is, the electrons from the conduction band trigger reduction reactions with energetically suitable acceptor species. The most commonly used semiconductor in photocatalysis is TiO_2_, not only due to its appropriate conduction band (CB) and valence band (VB) energy levels to react with many useful red‐ox partners such as H_2_O (to H_2_ and O_2_), but also its relatively large abundance, nontoxic nature, and maybe foremost due to its high photocorrosion resistance (the majority of common semiconducting materials including Si, GaAs, GaN, CdS, etc., have only a very limited resistance to photocorrosion). The bandgap of anatase TiO_2_ of ≈3.2 eV is the main drawback of the material, as it allows absorption only in the UV spectral range of sunlight. Despite this limitation, TiO_2_ and particularly its anatase form remain the benchmark for a semiconductor‐based photocatalyst—accordingly also it provides an ideal and stable model for elaborating concepts and mechanisms of photocatalytic reactions. There is a wide range of literature on the photocatalytic properties of TiO_2_, see for examples refs.[28, 29, 30, 31, 32, 33, 34, 35, 36].

For TiO_2_, excited electrons from the conduction band can react in a typical aqueous, aerated environment with H^+^ or O_2_ and reduce these to H_2_ and O_2_ **^·^** ^−^, respectively. These reactions do occur (are thermodynamically feasible), as the energetic position of the conduction band is higher than the redox potential of the targeted reaction. With the same reasoning, holes from the valence band can oxidize hole‐acceptor species, e.g., H_2_O to OH**^·^**, H_2_O_2_ or O_2_, or many organics finally to CO_2_ and H_2_O.^[^ ^28^ ^]^ As mentioned above, many hydrocarbons (alcohols, aldehydes) are much easier oxidized than water—therefore they often are used as sacrificial agents (i.e., they are used to capture holes before they can recombine with electrons). In terms of hole capture, the decomposition of organics to CO_2_ and water is a most efficient pollution degradation or self‐cleaning method (i.e., degrading organic dirt or environmental waste such as dyes and other undesired organic compounds to CO_2_ and H_2_O).^[^ ^37^ ^]^ More recently, more subtle targeted photocatalytic oxidation reactions are increasingly investigated for so‐called “upcycling,” i.e., to oxidize less valuable compounds to a targeted higher‐value oxidized compound.^[^ ^38^, ^39^, ^40^ ^]^ Overall, the main research direction of photocatalytic (and photoelectrochemical) approaches is still targeting water splitting (H_2_ generation), followed by pollutant degradation (self‐cleaning), and photocatalytic synthesis or upcycling.^[^ ^29^, ^37^, ^41^ ^]^

In the present review we first focus on how SA cocatalysts can be beneficial in the context of water splitting and we discuss according fundamental aspects, i.e., namely, the reaction of electrons with H^+^ in the water to form H_2_ and the accompanying oxidation of water or sacrificial agents. In Section 6, we then expand the discussion to other applications of SAs in photocatalysis.

Besides TiO_2_, in recent years particularly C_3_N_4_ is a strongly emerging semiconductor that has garnered significant attention.^[^ ^42^ ^]^ In contrast to TiO_2_ with a bandgap in the UV range (*E*_g_ = 3–3.2 eV), with a bandgap of ≈2.7 eV C_3_N_4_ is active under visible light irradiation, making it fundamentally more efficient in utilizing solar illumination.^[^ ^43^ ^]^ In many respects, C_3_N_4_ shows similar features to TiO_2_ (such as low cost, relative nontoxicity, and stable chemical nature). In addition, the electronic properties of C_3_N_4_ can be tuned by structural modification (via classic synthetic tools of organic chemistry), allowing—in principle—for great flexibility in optimizing its photon absorber photocatalytic performance.^[^ ^44^, ^45^ ^]^ Another unique feature of C_3_N_4_ is that its structure can provide a high number of potential coordination sites for anchoring metal precursors and many metal atoms.^[^ ^46^ ^]^ In the present review, we will therefore emphasize work that studies SAs on TiO_2_ and C_3_N_4_.

### 2.2 The Need for Cocatalysts

The activity of photocatalysts depends on the time scales of the involved photoinduced processes and the chemical reactions at the surface of the photocatalyst. For TiO_2_, this is well studied.^[^ ^29^, ^48^ ^]^ The formation of e^−^–h^+^ pairs upon illumination is fast, i.e., in the order of femtoseconds—the lifetime of electrons is in the range of 10–100 ns in rutile and a few ms in anatase. The lifetime of holes is in the range of 10–100 ns in both crystalline phases.^[^ ^49^ ^]^ Interfacial charge transfer typically occurs within the time interval of 10^−6^–10^−3^ s, i.e., the processes take place at a time scale similar to the recombination of e^−^–h^+^ pairs. Accordingly, often the majority of the photogenerated e^−^–h^+^ pairs (>90%) recombine after excitation rather than being transferred to a reactant in the environment. However, the kinetics of the interfacial charge transfer (and follow up red‐ox reactions) depends strongly on the nature of the semiconductor surface and the target reaction. For semiconductor surfaces such as TiO_2_, other oxides, and C_3_N_4_, many important charge transfer reactions are kinetically hindered. In particular, the classic HER and OER from water are slow on many semiconductive surfaces and therefore cocatalysts are used to accelerate the charge transfer reaction (Figure 3b). A most prominent example is the hydrogen production reaction that needs to be cocatalyzed using classic HER catalysts (as used in electrocatalytic applications) such as platinum, palladium, rhodium (i.e., elements at the tip of the classic Trasatti‐type volcano curves, see Figure 3c).^[^ ^47^ ^]^ These noble metals are often deposited as nanoparticles on the semiconductor surfaces.

In photocatalysis, there are two reasons for this beneficial effect of the cocatalyst. First the formation of a suitable electronic junction (Schottky junction) that is a metal contact on the semiconductor that facilitates electron transfer to the metal on the surface.^[^ ^50^ ^]^ The second beneficial effect is the promotion of the H_2_ formation reaction (Pt can catalyze the Volmer, Heyrovsky, and Tafel steps, i.e., the formation of H_ads_, the reaction of H_ads_ with solution H^+^, and the recombination of 2H_ads_ → H_2_).^[^ ^26^, ^47^ ^]^ To deposit the cocatalysts as nanoparticles (typically of some nm's diameter), various techniques based on PVD or on surface precipitation, impregnation, reduction, or photodeposition can be used.^[^ ^51^, ^52^ ^]^

Due to the high cost of noble metals (particularly when used on “cheap” semiconductors such as TiO_2_ or C_3_N_4_), there have been substantial research efforts to replace noble metals by cheaper species such as MoS_2_ or WSe_2_,^[^ ^27^ ^]^ by defect engineering,^[^ ^53^ ^]^ or by minimizing the amount of noble metals, where the ultimate minimum represents a single atom. The latter of course assumes that one atom of noble metal behaves at least as good as one nanoparticle. This is implying that scaling laws hold and thus assuming that one SA is as active as one NP, i.e., represents one catalytically active site.

Commonly, if TiO_2_ surfaces are decorated with a noble metal cocatalyst (e.g., Pt) of some nanometers in diameter, an acceleration of the charge transfer and the overall HER by a factor of 10–100 can be observed (Figure 3d). In view of a maximum gain by the use of SAs, the goal is to obtain at least the same improvement by the use of—as little as possible—noble metal SAs.

## 3 Features and Roles of SA Cocatalysts

### 3.1 General Features of SAs

The definition of what constitutes a “single atom catalyst” varies widely in literature. A wide body of literature essentially considers “active” atoms in some way attached to a substrate (as shown in Figure 2a)—and thus heterogeneous catalysts—as “true” SA catalysts.^[^ ^1^, ^2^, ^54^ ^]^ Nevertheless, in recent years catalytically active centers, e.g., in metalloorganic structures, aggregates, or molecules—and thus also homogeneous catalysts—are increasingly considered as SA catalysts, too.^[^ ^6^, ^7^ ^]^ The transition is somewhat fuzzy—in the present overview we focus primarily on substrate‐anchored SAs, with less emphasis on molecular structures containing catalytic atoms, such as photoactive organometallic compounds and homogeneous catalysts.

**Figure 4:**
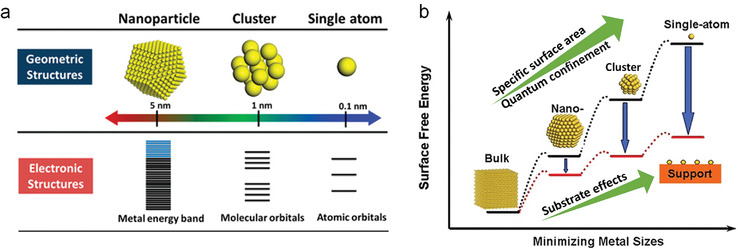
a) Geometric and electronic structures of single atom, clusters, and nanoparticles. b) Schematic illustrate the changes of surface free energy and specific activity per metal atom with metal particle size and the support effects on stabilizing single atoms. Reproduced with permission.^[^ ^1^ ^]^ Copyright 2013, American Chemical Society.

Some most attractive features of such SA‐substrate‐anchored catalysts are summarized in **Figure** **4**. The most mundane aspect is that if one atom indeed represents one active site, then the use of a minimum amount of precious metal means a tremendous financial incentive. Other aspects are scientifically certainly more fascinating, namely that the electronic properties of nanoscale particles change with particle size. Shrinking a catalytic particle down to few‐nanometer‐size clusters and further to a single atom, yields increasing quantum size effects—the smaller the stronger (from an electronic band structure of a conductive metal, to overlapping atomic orbitals of several distinct states to the isolated atomic orbitals of an SA). A secondary effect is that the smaller the nanoparticle, the more important become the ionic and electronic features of the substrate, i.e., substrate effects on the catalytic center become increasingly pronounced. All these effects may be used for altering/tuning the energetics of the catalytic center and thus enable unique or unusual reactions and to completely redirect a reaction path (e.g., strongly affect the selectivity of a reaction).

### 3.2 Changes of Optical Properties Caused by SAs

Loading of a single atom on a host semiconductor may lead to new electronic states in the bandgap of the semiconductor—this in turn can enhance the solar light absorbance. The effect of foreign atom loading or incorporation on the optical properties of TiO_2_ has been widely studied already in early work,^[^ ^55^, ^56^, ^57^ ^]^ where “doping” with species, e.g., of Pt, Fe, Cr, etc., were investigated in view of activating a visible response.^[^ ^56^, ^58^, ^59^ ^]^ A broad range of transition metals were reported to add intra bandgap states to TiO_2_ (or C_3_N_4_).^[^ ^56^ ^]^ In spite of these efforts, these doping effects remained of limited success, particularly due to the often observed deterioration of the electronic properties (electron mobility) in the doped material. Doping attempts were revitalized with the emergence of SA decorated semiconductors. Nevertheless, the question, if in practice there are truly beneficial effects depends on the absorption contribution of the SAs in comparison to the total light absorption in the semiconductor (i.e., the relative loading with SAs needs to be high). For example, Li et al.^[^ ^60^ ^]^ demonstrated the effect of Ru SAs decorated on monolayered TiO_2_ nanosheets. UV–vis diffuse reflectance spectroscopy of TiO_2_ nanosheets showed narrow optical absorbance with an edge at *λ* = 360 nm (across the TiO_2_ bandgap), attributed to the typical charge transfer from O 2p for valence band to Ti 3d for conduction band, whereas Ru‐decorated samples displayed broad light absorption up to *λ* = 700 nm. This was attributed to an additional charge transfer route from Ru d–d transition (shoulder peak ≈470 nm) and charge transfer to TiO_2_ states. Such optical effects of SAs on semiconductors are comparably widely discussed in Density functional theory (DFT) works^[^ ^61^, ^62^, ^63^, ^64^, ^65^, ^66^, ^67^ ^]^ and point out that metal SAs on semiconductors can enhance the visible light absorption ability. Note, the reported effect resembles in its core mechanism the classic Grätzel‐type Ru‐dye sensitization of TiO_2_.^[^ ^68^ ^]^ For C_3_N_4_, for example, Gao et al.^[^ ^61^ ^]^ calculated the optical absorption spectra of Pt‐ and Pd‐supported g‐C_3_N_4_, and found that with a Pd or Pt atom deposited on g‐C_3_N_4_, a new absorption peak appears at 0.7 eV and the absorption edge of g‐C_3_N_4_ extends from 2.7 to 0.2 eV. They ascribed this change to the electron excitation from the d band of metal atom to the conduction band of g‐C_3_N_4_.

However, while such calculations are interesting in terms of modifications of materials or surfaces and can point out ways to enhance the functionality of SA‐loaded semiconductor, the question is, if the result can be turned into a benefit for photocatalysis. This depends on a number of factors: first, the simple possibility to excite states (i.e., to absorb light) does not mean it is useful. Not only the energetic position of such states relative to the targeted red‐ox couples, but also the life‐time of the states, the extent of their localization (i.e., the coupling of such states with extended states of the semiconductor), i.e., classic parameters of semiconductor physics and electrochemistry, are decisive but often neglected.^[^ ^69^ ^]^ In other words, SAs decorated on semiconductor surfaces may in some cases provide visible light absorption, but the impact on practical applications is limited due to i) a lack of coupling of the states with the system (no mobility of generated charge carriers and as a result, no or very little photocurrent can be generated), and ii) a very high SA loading is needed to cause a sufficient effect on the overall light absorption.

### 3.3 Absence of Schottky Junction in SA Cocatalysts

A specific feature that is particularly noteworthy in the context of photocatalysis is that the usual description of a metal cocatalyst contact on a semiconductor needs to be reconsidered. The classic description of a noble metal particle on a semiconductor in terms of a Schottky junction is no longer reasonable.^[^ ^50^ ^]^ In classic Schottky junctions, the Fermi level of the semiconductor site is lowered to the Fermi level of the metal, due to the much higher available state density in the metals. As a result, a Schottky barrier is set up at the contact point with a depletion of charge carriers in the semiconductor.^[^ ^70^ ^]^ However, for small nanoclusters and SAs, this assumption is not valid since the few electronic states are not sufficient to alter the semiconductor charge on a considerable length scale.^[^ ^71^ ^]^ This means, for SA cocatalysts, a Schottky barrier as such does not exist, rather some electronic polarization which may be beneficial or detrimental for charge transfer depending also on the direction of the over‐the‐barrier electron transfer. Regarding the impact on the energy band structure, as discussed in Section 3.2, metal SAs primarily may be regarded more like a surface state (in classic semiconductor description), without significantly altering the intrinsic band structure. The exception to this would be if the metal atoms are inserted into the semiconductor lattice, in which case it could introduce intra bandgap states.

## 4 Critical Factors that Affect the Efficiency of SAs as Cocatalysts in Photocatalysis

### 4.1 Loading Density of SAs

**Figure 5:**
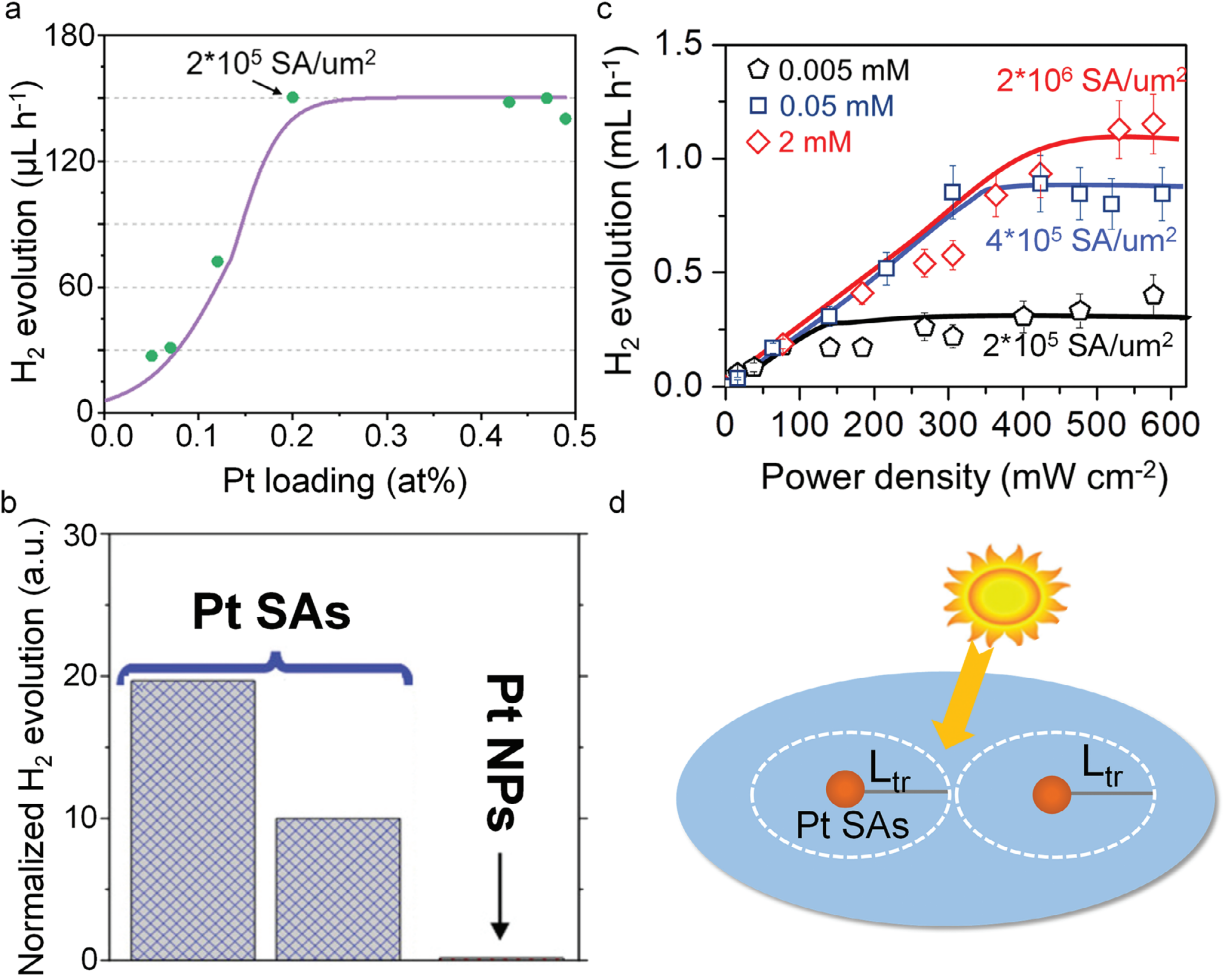
a) The effect of Pt loading on the photocatalytic H_2_ production activity. b) Comparison of photocatalytic H_2_ production activity using Pt SAs and Pt NPs as cocatalyst, respectively. c) Photocatalytic H_2_ evolution by the Pt SA‐loaded TiO_2_‐sputtered layer at different light intensities (365 nm LED) deposited in 0.005, 0.05, and 2 mm H_2_PtCl_6_ solutions, respectively. Reproduced with permission.^[^ ^72^ ^]^ Copyright 2023, American Chemical Society. d) Schematic of electron diffusion in the presence of Pt SAs. Reproduced with permission.^[^ ^72^ ^]^ Copyright 2023, American Chemical Society.

**Figure** **5**a illustrates the effect of Pt SAs on the photocatalytic H_2_ generation from TiO_2_. The results are from TiO_2_ compact layers (anatase, 200 nm thick) that are loaded with different amounts of Pt SAs (more precisely, an increasing density of Pt SAs). Increasing the Pt loading leads to an increase of the hydrogen generation activity, but only up to a certain level. Saturation occurs—in the given example—at a concentration of ≈0.2 wt% platinum (≈2*10^5^ SAs per µm^2^) on the TiO_2_ surface. At higher concentrations no further increase in the H_2_ evolution reaction is observed. This indicates that for this particular TiO_2_ semiconductor and illumination, the critical cocatalyst density needed to utilize all photogenerated electrons is reached at 2*10^5^ SAs per µm^2^, i.e., this point represents optimized Pt loading—a further increase in Pt loading is not necessary for maximizing H_2_ production efficiency—the Pt is wasted. Comparing at this point the efficiency of Pt SAs with Pt nanoparticles, the mass‐normalized H_2_ evolution activity of Pt SAs is ≈100–1000 times more effective than that of Pt NPs (Figure 5b).

In classic catalysis concepts, a high density of catalytic sites is usually demanded, as this directly determines the rates of many reactions—as a consequence numerous studies in SAC design focus on increasing the loading amount (surface density) of SAs.^[^ ^73^, ^74^, ^75^ ^]^ This is not the case for photocatalysis. Several recent studies show that a low loading of metal SAs is sufficient for photocatalytic H_2_ production for many semiconductors and considering solar irradiation. For example, a cyanide leaching experiment on Pt‐loaded TiO_2_ showed that the removal of over 90% of the Pt on TiO_2_, after standard Pt deposition,^[^ ^76^ ^]^ does not lead to a loss of activity, i.e., neither Pt^0^ species (metallic nanoparticles) nor the majority of Pt SAs are needed to provide the cocatalytic activity of platinum on TiO_2_. Such conclusions can also be drawn from investigations of the influence of SA loading and applied light intensity on photocatalytic H_2_ production activity.^[^ ^72^ ^]^ The results show that under the vast majority of common illumination conditions and already at a low SA loading, the rate‐determining factor is not the density of cocatalytic sites (as shown in Figure 5a) but rather the generation and collection of charge carriers and thus the flux of photoelectrons to the cocatalytic centers. This finding also applies to other semiconductors than TiO_2_, such as C_3_N_4_. A low loading amount of Pt SAs on C_3_N_4_ is sufficient to achieve optimal photocatalytic hydrogen production efficiency.^[^ ^72^, ^77^ ^]^

### 4.2 Illumination Intensity of Light (Light Absorbance)

In general, a high light intensity results in the generation of more electron–hole pairs,^[^ ^78^, ^79^ ^]^ which can increase the photocatalytic reaction rate—this if sufficient catalytic (electron transfer) sites are provided. The connection of light intensity, other typical semiconductor parameters (absorption coefficient, carrier mobility, diffusion length), intrinsic and extrinsic fields, as well as the generation and flux of photogenerated charge carrier is provided in the classic semiconductor electrochemistry literature, i.e., the works of Gärtner and Butler,^[^ ^78^, ^79^ ^]^ Johnson and Tauc,^[^ ^80^, ^81^ ^]^ Mott and Davies,^[^ ^82^ ^]^ and Gerischer and Marcus.^[^ ^83^, ^84^ ^]^

Accordingly, the photocatalytic rates may be determined either by such charge carrier generation aspects or by charge carrier transfer that may be represented by the number density of transfer sites (SA density) and their activity (i.e., their individual turn over frequency, TOF). Figure 5c illustrates this—shown is the H_2_ evolution efficiency as a function of light flux, with illumination power‐densities ranging from ≈1 to 600 mW cm^−2^ for three different Pt SA loadings on compact TiO_2_ layers. At lower light intensities, up to ≈100 mW cm^−2^, H_2_ production increases linearly with higher power density for all loadings. In this range (which corresponds to ≈0–10 sun illumination), the H_2_ production rate is primarily determined by the light‐harvesting properties of the substrate, i.e., by the optical and electronic properties of the semiconductive substrate.

Beyond a certain illumination threshold, further increase in light intensity leads to no further activity increase due to a lack of available cocatalytically active sites. As shown in Figure 5c, saturation occurs at a light intensity of 140 mW cm^−2^ for a density of 2 × 10^5^ µm^−2^ Pt SAs. For a density of 4 × 10^5^ µm^−2^ Pt SAs, saturation is observed at 370 mW cm^−2^, and for 2 × 10^6^ SAs per µm^2^ it occurs at 450 mW cm^−2^.^[^ ^72^ ^]^ This represents a TOF of ≈200 H_2_ molecules per site per second. While the specific values depend on the system studied, the general features are common to photocatalytic systems. Roughly a loading of 10^5^–10^6^ SA per µm^2^ is sufficient under solar light to reach a maximum cocatalytic effect (if the electronic properties of the substrate are reasonable).

### 4.3 Optimal SA Design

Above discussion emphasizes the critical role of semiconductive properties and loading density in determining the efficiency of Pt SAs as cocatalysts. To achieve optimal efficiency, these factors must be considered for a specific substrate (namely, light absorption, mobility of photoexcited carriers, surface recombination rates) and possible other rate‐limiting reactions (such as diffusion effects in the liquid phase). In general, the “better” the quality of the semiconductor, the lower the required SA density.

An ideal placement of electron transfer mediators may, in a most simple approach, be approximated by using a spacing of mediators (cocatalysts), as illustrated in Figure 5d with a spacing of 2L_ec_ (where L_ec_ is the electron surface collection length). This, if electron transfer and reaction (as opposed to hole transfer) is the rate‐determining step. These considerations illustrate the high potential of an optimized SA loading toward a minimized number of active sites for achieving a maximized reaction rate. In the example of Pt SAs on the TiO_2_ surface of Figure 5a, 1 Pt atom per 10 nm^2^ is sufficient to deal with all arriving electrons for maximized H_2_ production efficiency. This coverage corresponds to a Pt cost of ≈0.1–0.2 cent m^−2^; note, this makes Pt an economically viable option for photocatalysis. It also should be mentioned that the L_ec_ in many semiconductors and at many semiconductor electrolyte interfaces is significantly smaller than the bulk electron diffusion length, as surface defects or other surface states (e.g., solution induced) strongly affect L_ec_.^[^ ^72^ ^]^

Above discussion on L_ec_ assumes that the supply of charge carriers to the reactive center, the cocatalyst SA, is rate‐determining (and accordingly an ideal descriptor for SA spacing is provided by the surface electron collection length). In some cases, this may not be the appropriate descriptor, namely, when “spillover” effects determine the reaction rate.

Hydrogen spillover is a well‐documented phenomenon in catalysis, particularly in heterogeneous catalysis and hydrogen storage.^[^ ^85^, ^86^ ^]^ It describes the migration of hydrogen atoms (H) or protons (H⁺) from a metal catalyst—where hydrogen dissociation occurs—to an adjacent support material, which is typically less effective at dissociating H_2_ but may provide active sites for further reactions. In such cases, an optimum spacing would be determined by the spillover‐length L_spill_, i.e., the spillover active zone around an SA. In literature on photocatalytic H₂ production, however, the primary focus is on the generation and subsequent reactions of light‐induced electron–hole pairs rather than on hydrogen spillover mechanisms. Since the reaction is perceived to predominantly take place at the metal cocatalyst sites, hydrogen spillover to other regions of the catalyst is generally neglected, as photocatalysis usually occurs at ambient or lower temperatures, where the activation energy for H_2_ spillover is low. Nevertheless, some studies have reported evidence of H_2_ spillover in photocatalytic and photoelectrocatalytic H_2_ production,^[^ ^87^, ^88^, ^89^ ^]^ For example, hydrogen dissociation and spillover on Au/TiO_2_ has been explored using Infrared (IR) spectroscopy,^[^ ^89^ ^]^ but since these characterizations were not conducted under true photocatalytic conditions, the validity of the H_2_ spillover mechanism in these systems remains uncertain. Additionally, studies on metal SAs decorated on semiconductors have suggested that metal SAs can promote H_2_ dissociative adsorption.^[^ ^90^, ^91^, ^92^ ^]^ However, results on spillover are mainly based on theoretical models—a main reason for this is that experimental access to spillover length is difficult and mostly limited to modified scanning probe microscopy techniques.^[^ ^93^ ^]^

#### 4.3.1 Turnover Frequency (TOF)

TOF is a metric adopted from classic catalysis, representing the number of catalytic cycles a single active site can perform per unit time. The upper limit of a Pt atom to convert water to hydrogen in an electrochemical reaction is still not finally explored but estimated to be in a range of TOF ≈ 10^2^–10^4^ site^−1^ s^−1^.^[^ ^94^ ^]^ In photocatalytic experiments, these values can be reached at low loading and high illumination intensities.^[^ ^72^ ^]^ For photocatalysts, TOF is not often considered a primary descriptor for performance evaluation; instead, metrics such as apparent quantum yield (AQY) and solar‐to‐hydrogen (STH) efficiency are commonly used.^[^ ^95^ ^]^ This focus arises because semiconductor parameters frequently are (and need to be) emphasized, rather than the rate‐determining step at the cocatalyst. However, when evaluating the efficiency of metal SAs as cocatalysts, it is essential to consider the TOF, and particularly the maximum TOF of a site (i.e., the maximum rate of molecules (e.g., H_2_) that can be produced per active site—particularly when the conditions are such that the maximum TOF can become rate‐determining: low SA loading, high charge carrier flux, strongly concentrated light.

A common practical issue is the determination of the SA surface density used for the evaluation of TOF from an experiment. In many cases, when powdered photocatalysts are used, an evaluation of the TOF can become cumbersome—there are excellent discussions about this issue in literature.^[^ ^96^, ^97^ ^]^ In order to avoid these issues, experimental models that operate with defined conditions and substrates (e.g., flat thin films that allow a direct count of the SA density from high‐angle annular dark‐field scanning transmission electron microscopy (HAADF‐STEM) and defined constant illumination) have become very helpful.^[^ ^98^, ^99^, ^100^ ^]^

#### 4.3.2 Specific Surface Location of SAs “Hot Spots”

An important point for the activity (and stability) of SAs is an optimum placement, i.e., a most effective position on the surface (in the lattice) and its coordination. While such superactive SA sites are highly desired, experimental access and creation is still on an early level of understanding, e.g., Pt nanoparticles and SAs deposited on (101) surfaces of TiO_2_ are stabilized and more reactive than placed on (001) surfaces.^[^ ^101^ ^]^ Remarkable work of Christopher and co‐workers showed the importance of Pt placement on TiO_2_ surfaces. They found that Pt atoms on the terraces of the TiO_2_ (101) surface are in a highly stable coordination state.^[^ ^102^, ^103^ ^]^ This was established through a series of experiments, where they employed a synthesis approach to achieve an average of <1 Pt atom per TiO_2_ particle, ensuring that the atoms remained mobile meanwhile without forming clusters. In subsequent work,^[^ ^104^ ^]^ they applied this approach to Pt SAs on shape‐controlled TiO_2_, specifically on (001)‐ and (101)‐dominated TiO_2_ surfaces. The results showed that Pt SAs exhibit higher reactivity for CO oxidation on (101) surfaces compared to (001) surfaces. More recently, it was shown that SA Pt can be accumulated on (101) surfaces of anatase crystallites and these SAs provide enhanced reactivity and stability.^[^ ^105^ ^]^

For C_3_N_4_, various experiments and theoretical calculations indicate that metal atoms preferentially coordinate with nitrogen atoms, forming stable metal–nitrogen (M–N*_x_*) coordination bonds. Among these, the most stable configuration is achieved when the metal atoms are anchored at the N_4_ coordination sites, where they form strong interactions with four adjacent nitrogen atoms.^[^ ^62^, ^106^, ^107^ ^]^ This M–N_4_ coordination state has been reported in many metal SAs decorated C_3_N_4_ systems (discussed in Section 6).

As a result, it is clear that SAs on surfaces are not “equal”—there are “active,” “hot spots” that are ideally coordinated and less active or inactive ones. This optimal positioning in photocatalysis may not only be determined by the optimized chemical coordination but also in view of preferred electron exit sites.^[^ ^105^ ^]^

Another example may be the work of Chang et al. who used aberration‐corrected STEM to characterize Pt SAs on TiO_2_ (110) surface and to identify different adsorption sites for Pt atoms on the TiO_2_ surface.^[^ ^108^ ^]^ Combining their observations with DFT calculations, they concluded that the most favorable adsorption sites for Pt are the vacancy sites of basal oxygen atoms located in subsurface positions relative to the top surface bridging oxygen atoms. However, this conclusion was contradicted by later computational work by Thang et al.,^[^ ^102^ ^]^ which showed that structural models with oxygen vacancies cause a red shift in CO adsorption energy, inconsistent with the observed features of isolated Pt species. Notably, different TiO_2_ surfaces were examined in these studies—(110) in the former and (101) in the latter—making direct comparison and conclusions uncertain.

Surface placement of SAs can strongly depend on the selected synthesis reactions where statistical or site‐selective placement may be achieved.

In addition to the surface coordination of one single atom, also the interactions among SAs—including the formation of dimers, trimers, and few‐atom clusters—are frequently debated and remain a topic of controversy.

For instance, while some studies suggest that SA dimers and trimers can enhance catalytic activity by creating synergistic effects, others argue that these larger assemblies may undermine the unique properties of isolated SAs. Few‐atom clusters, in particular, are seen by some researchers as beneficial intermediates between SAs and bulk metal particles, potentially improving stability and activity. However, the significance of small assemblies up to few‐atom nanoclusters at present seems unclear, and clearly further and more detailed studies are needed to clarify relevant effects for photocatalysis.^[^ ^109^, ^110^ ^]^

## 5 Synthesis, Stabilization, and Characterization of SAs

**Figure 6:**
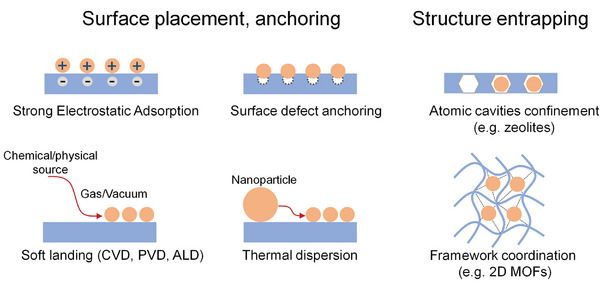
Schematic illustrations of techniques to deposit SAs on substrate surfaces.

A large number of synthesis approaches of SACs have been described in detail also in various reviews.^[^ ^13^, ^20^, ^54^, ^111^, ^112^, ^113^, ^114^ ^]^ **Figure** **6** gives an overview over most typical techniques—they widely involve wet immersion techniques,^[^ ^115^ ^]^ or gas phase physical or chemical approaches.^[^ ^116^, ^117^ ^]^ A homogeneous dispersion of SAs and a controllable loading amount are critical for providing efficient SACs. A critical point in all synthesis is that due to the high surface energy of SAs, there is a significant tendency for individual SAs to aggregate into clusters or nanoparticles either during deposition or during application in the reactive environment. It is therefore necessary to stabilize the SAs on the support to hamper or prevent aggregation. A common pathway to directly produce chemically or sterically stabilized SAs is to entrap them in atomic cavities such as provided in zeolites or in MOFs.^[^ ^118^ ^]^ Other approaches use chemical structural configurations, for example, by organometallic coordination (often a combination of light absorber and SA catalysts such as in 2D MOFs).^[^ ^119^, ^120^ ^]^ These structures typically exhibit highly uniform chemical ordering and surface properties. While such entrapping measures provide a high degree of stabilization of SAs, they often limit access of reactants to the reactive center and—particularly in photocatalysis—block the access of light and/or photogenerated carriers to the reactive center.

In the case of attaching SAs onto surfaces, mainly surface defects play a key role in immobilization; such defects may be vacancies, steps, grain boundaries, dislocations, adatoms, or foreign surface embedded atoms.

A full range of other less commonly used SA deposition processes are provided in the literature.^[^ ^121^, ^122^, ^123^, ^124^, ^125^ ^]^ Maybe a most interesting counterintuitive approach is thermal dispersion of clusters to SAs (opposite to commonly expected thermal agglomeration)—however, if this process is possible or not depends strongly on the substrate.^[^ ^122^, ^123^ ^]^

Traditionally, SAs were deposited by high vacuum deposition techniques, such as size‐selected atom and cluster deposition.^[^ ^126^ ^]^ These techniques typically are based on atomic or ionized species that then are size selected by a mass spectrometric filter (to select nanoclusters according to mass) and then at low energy (soft landing) deposited on substrates. This allows to deposit metal clusters with a precise number of atoms in ultrahigh vacuum (UHV) and under very defined conditions.

More recently, atomic layer deposition (ALD), as a typical vapor‐phase deposition technique has attracted increasing attention. In ALD, in a pulse process precursor molecules (metal–organic compounds) are chemically adsorbed on the support surface forming an atomic adsorbate monolayer. To deposit SAs, the ALD process is typically controlled to deposit sub‐monolayers of the precursor while the organometallic coordination is decomposed. Conceptually, it is a well‐defined method that allows for precise control over the loading of single atoms, dual atoms, or small clusters on a substrate. By optimizing the ALD conditions, SAs deposition can be achieved for a wide range of catalytic materials.^[^ ^116^ ^]^

Most common in materials science are solution‐based techniques (wet synthesis approaches) that are simply based on the immersion of a substrate into a suitable precursor solution—surface attachment then relies on two principles: precursor adsorption and/or precursor reaction. A basis for a controlled adsorption is the concept of strong electrostatic interaction—it allows for a well dispersed adsorption of precursor complexes that later are thermally ashed (burn off the ligands) to yield surface metal atoms.^[^ ^127^ ^]^ Alternatively, a defined chemical reaction of the precursor with the substrate can take place leading directly to SA attached on a surface (at distinct reactive surface locations), this then is termed “reactive deposition.”^[^ ^128^ ^]^

In view of their wide use, we discuss below the most relevant solution‐based approaches in more detail.

### 5.1 Wet Synthesis Approaches for SAs

Solution based techniques for the fabrication of classic catalysts on a support—as well as SAs on support—depend on several steps in the preparation, including the support composition, metal salt, method of metal addition, pH, metal loading, calcination temperature, etc. They all affect the ultimate loading and particle size of the final catalyst. So‐called incipient wetness or dry impregnation is often used in the preparation for Pt‐based catalysts, e.g., using chloroplatinic acid (H_2_PtCl_6_), or platinum tetraamine chloride, Pt(NH_3_)_4_Cl_2_.^[^ ^129^, ^130^, ^131^, ^132^ ^]^ With this method, the desired amount of metal salt is dissolved in sufficient water (or solvent) to just fill the pore volume of the support, i.e., no Pt is wasted in the solution. More often, the precursor and the substrate (powder) are mixed to form a slurry with excess solution. This method is commonly called ion exchange, or wet impregnation.^[^ ^133^, ^134^ ^]^ This method to a large extent relies on the electrostatic interaction (adsorption) of the precursor complex with the substrate.

#### 5.1.1 Strong Electrostatic Adsorption (SEA)

**Figure 7:**
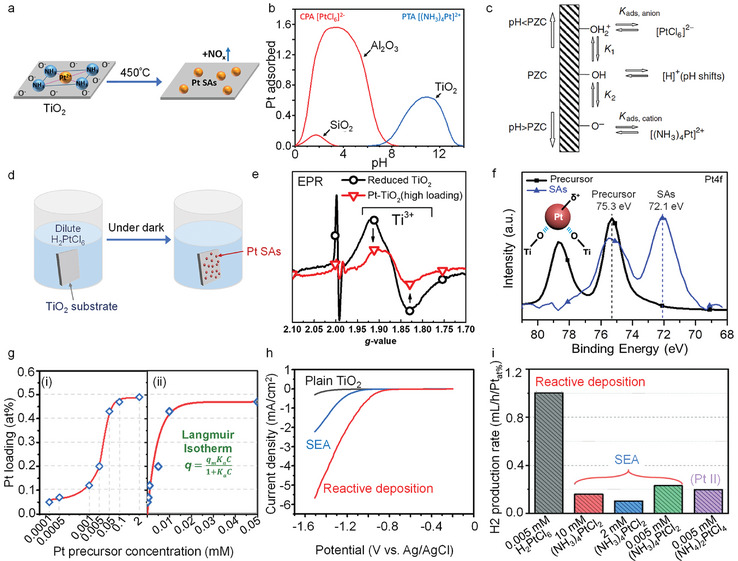
a) Schematic illustrations of SEA method for Pt SAs deposition on TiO_2_. b) Revised physical adsorption prediction of Pt adsorption over SiO_2_, Al_2_O_3_, and TiO_2_. c) Electrostatic adsorption mechanism showing protonation–deprotonation of surface hydroxyl groups on oxide surfaces at PZC and the resulting uptake of anionic or cationic complexes, influencing solution pH. Reproduced with permission.^[^ ^141^ ^]^ Copyright 2006, Taylor & Francis Group. d) Schematic illustrations of reactive deposition method for Pt SAs deposition on TiO_2_, and e) Electron paramagnetic resonance (EPR) spectra of reduced TiO_2_ before and after Pt SAs deposition. Reproduced with permission.^[^ ^98^ ^]^ Copyright 2020, Wiley‐VCH. f) Pt 4f XPS spectra of H_2_PtCl_6_ precursor and Pt SAs on TiO_2_ substrate. g) (i) The relationship between Pt loading and the Pt precursor concentration. (ii) The correlation between low concentration of Pt precursor and Pt loading. h) LSV curves of bare TiO_2_ anatase layers, and after Pt SAs deposition via reactive or SEA approach. Reproduced with permission.^[^ ^128^ ^]^ Copyright 2023, Wiley‐VCH. (i) Comparison of photocatalytic H_2_ production performance of Pt SAs decorated on TiO_2_ by reactive deposition method and strong electrostatic adsorption method. Reproduced with permission.^[^ ^128^ ^]^ Copyright 2023, Wiley‐VCH.

Strong electrostatic adsorption (SEA) is the most widely used approach to achieve maximum dispersion of noble metals on many oxides (such as Al_2_O_3_, SiO_2_, TiO_2_).^[^ ^127^, ^135^, ^136^, ^137^, ^138^, ^139^, ^140^ ^]^ The approach was originally developed by Brunelle,^[^ ^139^ ^]^ Conţescu and Vass,^[^ ^140^ ^]^ and Regalbuto and co‐workers^[^ ^137^, ^138^ ^]^ —it is illustrated in **Figure** **7**a–c —and the technique laid the fundamentals for the preparation of modern supported catalysts and up to today is considered to be the key strategy in wet noble‐metal catalyst loading on many support surfaces. For oxide surfaces underlying this strategy is that hydroxyl groups present on the surfaces in aqueous solutions become deprotonated and thus negatively charged at pH value above the point of zero charge (PZC).^[^ ^141^ ^]^ As a consequence, the surface would strongly adsorb cations such as tetraamine platinum, ((NH_3_)_4_Pt)^2+^ in an alkaline environment. In acidic environments, the oxides get protonated (and positively charged), i.e., anions such as PtCl_4_^2−^ are strongly adsorbed in acidic environment. Further catalyst preparation usually consists of a washing step and importantly followed by thermal post‐treatments to achieve the active (precursor‐ligand‐free) and anchored entity.

The uptake‐pH curve for charged metal complexes on many types of oxides shows typically a maximum (see Figure 7b). It lies at a point where the pH yields a maximum surface ionization (and thus charge), see scheme in Figure 7c, and at the same time is not so high that the solution ionic strength shields the attractive charge for precursor complex adsorption. This situation leads to a maximum in the amount of adsorbed of charged metal complexes.

Although conceptually simple and straightforward, in practice, this approach works well for certain oxides such as Al_2_O_3_, SiO_2_, and TiO_2_ but usually only one of the two pathways is successful (this is ascribed with their specific PZC, complex speciation, ionic strength, and coadsorption effects, however commonly a maximum loading is obtained at pH 3–4 for SiO_2_, at pH 8–9 for Al_2_O_3_ and at pH 9–12 for TiO_2_).^[^ ^138^, ^141^, ^142^, ^143^ ^]^

One key reason for practical variations from the concept is the hydrolysis effect on the precursor complex speciation in solution, i.e., a range of hydrolyzed species with various charge are present in solution (and speciation is influenced by pH and ionic strength). All these factors affect adsorption on a surface from a solution. As a result, many deposition recipes are subject to educated trial and error. A well investigated example is H_2_PtCl_6_ speciation that varies depending on pH and ionic strength—adsorption may involve the entire PtCl_6_^−2^ ions or a partially aquated/hydrolyzed form, such as Pt(H_2_O)*_x_* OH*_y_* Cl*_z_^n^* ^−^. For some oxides, coupling or grafting onto oxygen sites may occur.^[^ ^144^ ^]^ Moreover, H_2_PtCl_6_ is partially prone to self‐reduction, where the splitting off of Cl_2_ or HCl from attached precursors has been postulated.^[^ ^145^ ^]^ Moreover, reactions during post‐treatments at elevated temperatures can depend strongly on the initial adsorption pathway.^[^ ^146^ ^]^ Consequently, achieving a high level of Pt dispersion and the feasibility of obtaining single‐atom dispersion strongly depends on the precise elaboration of these parameters.

#### 5.1.2 Reactive Deposition

In contrast to the SEA concept, some recent works report on a reactive deposition method (namely, on TiO_2_) using highly diluted (µm concentration) H_2_PtCl_6_ solutions (Figure 7d).^[^ ^128^ ^]^ In the process, a surface reaction reduces the Pt^4+^ of the precursor to surface bound Pt^δ+^ SA species where *δ* ≈ 2, i.e., in this case, a reductive (thus reactive) deposition of Pt on TiO_2_ takes place and leads to uniformly dispersed Pt SAs. Remarkable is that the resulting Pt SAs have shown to be very effective for photocatalytic H_2_ generation, as discussed in Section 5.13.^[^ ^15^, ^72^, ^100^, ^128^, ^147^ ^]^

The reactive deposition process is based on surface defects in TiO_2_, almost any form of TiO_2_ contains oxygen vacancies and Ti^3+^ states either native or intentionally introduced (e.g., by chemical or thermal reductive treatment in H_2_ or Ar/H_2_). Such defects are particularly well apparent in Electron paramagnetic resonance (EPR) spectra (Figure 7e). The broad signal in the range of g‐values of 1.85–1.95 is typical of Ti^3+^ surface defects in TiO_2_.^[^ ^148^ ^]^ After exposure to a H_2_PtCl_6_ solution, the defect signature shrinks significantly which reflects a galvanic reaction where the Ti^3+^ states are oxidized to Ti^4+^ and Pt^4+^ in the precursor is reduced to Pt^2+^ on the surface.

In X‐ray photoelectron spectroscopy (XPS) the surface reaction is apparent from the Pt 4f signal, i.e., for Pt^4+^ (precursor) with a Pt 4f doublet at 75.3 and 78.7 eV—after reaction Pt^2+^ is surface attached with Pt 4f at 72.1 and 75.5 eV (Figure 7f). At the same time, the chlorine XPS signal is entirely lost—this shows that the precursor has fully reacted off and Pt is no longer Cl‐coordinated in the attached state. In this situation Pt is present as Pt^δ+^ with *δ* ≈ 2 and corresponds to an oxygen‐coordinated Pt SA as shown in the inset.

Figure 7g shows the Pt loading of a TiO_2_ surface with increasing precursor concentration. For increasing precursor concentrations using H_2_PtCl_6_ solutions from µm to the mm range, the loading first increases but saturation is reached already at ≈0.01 mm Pt solution, corresponding to 0.5 at% Pt on the surface. Higher precursor concentrations under these conditions do not lead to a further uptake of Pt. The Pt‐uptake follows a Langmuir type of adsorption characteristics, which reflects the presence of a finite amount of docking sites (reactive sites) that allow Pt SA attachment on this specific type of TiO_2_ surface (Figure 7g‐ii).

If these TiO_2_ samples with different Pt loading are tested for H_2_ evolution under near solar illumination conditions, a saturated activity is obtained already at a Pt loading of 0.2 at%, see Figure 5a, i.e., although reactive deposition allows only for a relatively low surface loading, this Pt decoration is entirely sufficient to maximally cocatalyze H_2_ production. This sort of reactive deposition has been reported for a wide range of TiO_2_ morphologies, including nanotubes, powders, flat layers, single crystals, as well as for other semiconductors such as C_3_N_4_.^[^ ^15^, ^99^, ^100^, ^128^, ^149^, ^150^, ^151^, ^152^ ^]^ Most importantly, the reactive deposition seems to provide “self‐homing” features as described below.

#### 5.1.3 The Self‐Homing Principle: Positioning of Single Atoms for Maximum Efficiency

Considerable work shows that the exact surface anchoring sites on a substrate can strongly affect the reactivity of a surface placed atom, in classic catalysis as well as in photocatalysis, see also Section 4.3.2.

In general, real surfaces have unsaturated sites, including point defects, inclusions, step edges and other specific surface configurations that may be particularly reactive for a specific reaction—these sites may represent a “catalytic hot‐spot.” They may also be specifically active for the attachment of noble metal species (e.g., single atoms, nanoparticles) as has been classically widely employed in “sensitizing” surfaces for galvanic reactions.^[^ ^153^ ^]^ If, following attachment, the noble metal species attain a highly reactive state—sometimes referred to as “super‐reactive” or a “hot spot” configuration—this attachment process can be described as “self‐homing.”

Reactive deposition is a process that seems to be of a “self‐homing” nature (i.e., deposition of SAs takes place at the location where they are most effective). That is, deposition takes place at most reactive surface locations, and after deposition this SA decorated location represents again a “hot spot” for a target reaction. Reactive deposition thus leads to particularly active spots, while statistical deposition leads to less active configurations. This can be well illustrated when comparing SAs deposited by SEA and by reactive deposition. Figure 7h shows electrochemical Linear sweep voltammetry (LSV)Nulcear curves taken for TiO_2_ surfaces that were loaded with the same density of SAs, once with SEA leading to a random Pt SA distribution, once with Pt reactive deposition.^[^ ^128^ ^]^ In the *I*–*V* curves, the HER onset and activity are significantly different. Evidently the SAs from the surface with reactive deposition show much higher current densities (activity of the SAs). This implies that the surface anchoring of these SAs occurred at more favorable spots for electron transfer in comparison to the random deposition from SEA. Further evidence of the effectiveness of the reactive deposition method is provided by the comparison of photocatalytic H_2_ production between Pt SAs decorated on TiO_2_ using reactive deposition and the SEA method, see Figure 7i.^[^ ^128^ ^]^ It is clear that the reactive deposition method results in significantly higher efficiency of Pt SAs in photocatalytic H_2_ production, even with a low amount of Pt precursor. In contrast, regardless of the concentration of Pt precursor used, the efficiency of Pt SAs in the SEA case remains much lower.

The self‐homing feature describes that dilute Pt‐acid precursors naturally react at the most reactive sites on a substrate (until they are saturated). This selective reaction process leads then to Pt SAs in a most active surface configuration. These self‐anchored SAs are precisely positioned at these reactive sites, and therefore can create exceptionally active cocatalytic centers that significantly enhance electron transfer processes.

This principle not only contributes to the superior performance of the photocatalyst but also plays a vital role in its high dispersion. SAs that are anchored where they are most effective eliminates the need for excessive loading, the self‐homing principle thereby reduces material waste and potential agglomeration issues. In essence, the self‐homing principle emerges as the key factor in achieving a highest possible efficiency and stability in SA‐based photocatalysis systems.

Please note that Pt utilization efficiency can further be maximized it SAs are concentrated (on semiconductor junctions) only to electron exit sites (see Section 4.3.2).

### 5.2 Agglomeration of SAs

**Figure 8:**
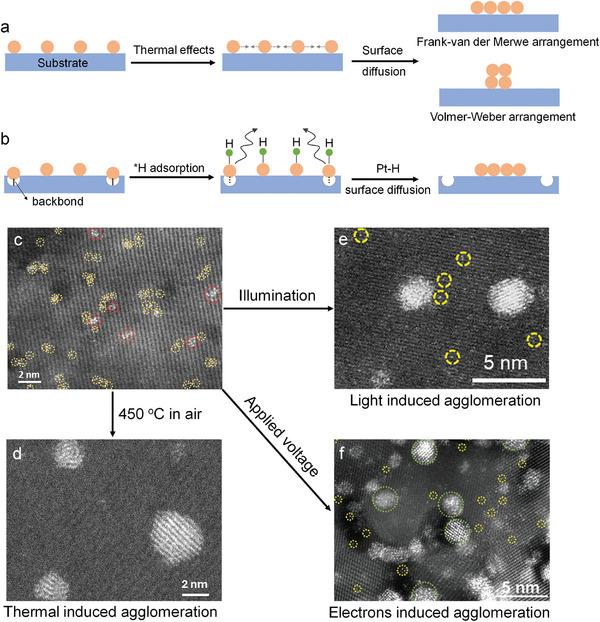
a,b) Schematic illustration of SA agglomeration under (a) thermal treatment, and (b) catalytic conditions. c–f) HAADF‐STEM image of (c) Pt SAs decorated TiO_2_, (d) Pt SAs decorated TiO_2_ after thermal treatment, and (e) Pt SAs decorated TiO_2_ after light illumination. Reproduced with permission.^[^ ^152^ ^]^ Copyright 2023, Wiley‐VCH. (f) Pt SAs decorated TiO_2_ after electrocatalytic HER. Reproduced with permission.^[^ ^99^ ^]^ Copyright 2024, Wiley‐VCH.

The stability of SAs under thermal load or catalytic working conditions is one of the most critical concerns in SA catalysis (**Figure** **8**).

Under classic thermodynamic considerations to form (maintain) stable SAs, their chemical potential should be lower than in typical metal NPs. This may be reached by surface‐, ligand‐, or electronic effects. In such cases, in fact thermal dispersion of NPs can be achieved.^[^ ^154^ ^]^ If agglomeration takes place, then for the formation of M–M bonds there are various classic possibilities, again depending on the substrate interactions. For example, if a substrate has stronger interactions than the adatoms among each other (they favor a Frank–van der Merwe arrangement as opposed to a Volmer–Weber arrangement) and 3D agglomeration can be largely prevented.^[^ ^155^, ^156^ ^]^ Such classic nucleation theories and buildup of critical size cluster stability sites can be applied, but the specifics of SA agglomeration to few‐atom 3D clusters or 2D rafts require careful analysis and experimental considerations (Figure 8a).

In general, atomic diffusion, aggregation, and disintegration during catalyst preparation occur via thermal effects, such as Ostwald ripening, leading to the growth of larger NPs or 2D assemblies that eventually reach thermodynamic equilibrium.^[^ ^157^ ^]^ These are considerations for thermally induced agglomeration and dispersion. In photocatalysis, except for thermal agglomeration (pre‐ or post‐treatments of photocatalysts), there is also so‐called “light‐induced agglomeration.” This term is somewhat misleading as it generally describes agglomeration under photocatalytic H_2_ generation conditions,^[^ ^152^ ^]^ but it also occurs under electrocatalytic HER.^[^ ^99^ ^]^ In fact, this agglomeration is actually caused by the formation of a reaction intermediate that destabilizes the bond of the SA to the substrate. As illustrated in Figure 8b, when a semiconductor decorated with metal SAs (e.g., Pt SAs on an oxygen vacancy in TiO_2_) is used for photocatalytic or electrocatalytic HER, hydrogen intermediates (*H), and as a result Pt–H intermediates are formed. DFT shows that this interaction weakens the anchoring of Pt to the substrate, thus making the Pt SAs (or more precisely the Pt–H intermediate) mobile.^[^ ^152^ ^]^ As a result, they may migrate and agglomerate into a more thermodynamically stable state forming Pt clusters and/or nanoparticles.

Figure 8c,d provides examples of thermal‐induced, “light‐induced” and “electrochemically induced” agglomeration.^[^ ^99^, ^152^ ^]^ Figure 8c shows a TiO_2_ surface loaded with Pt SAs. After annealing at 450 °C in air, large Pt nanoparticles are observed in HAADF‐STEM image (Figure 8d). Similar agglomeration behavior occurs under light illumination^[^ ^152^ ^]^ and under electrocatalytic conditions,^[^ ^99^ ^]^ where Pt nanoparticles can be seen within minutes of light illumination (Figure 8e,f). This is confirmed by the Pt 4f XPS spectra, which show that Pt^δ+^ species are to a large extent reduced to metallic Pt^0^ after a few minutes of light exposure.

Therefore, strong bonding is essential to obtain long‐term photocatalytic stability. There are several types of anchoring sites used to form SAs: i) defects on the support material, ii) unsaturated coordinated atoms, and iii) cavities or cages inherent to the material's structure.^[^ ^54^ ^]^ In Section 5.3, the practical aspects of stabilization of SAs will be discussed in more detail.

### 5.3 Stabilization Strategies of SAs

#### 5.3.1 Heat Treatments

Heat treatments in SA catalyst preparation are widely used to i) eliminate coordinated ligands from an adsorbed precursor (wet techniques, ALD, etc.), ii) reduce ionic forms of precursors to metals, and iii) to stabilize SAs in specific surface locations (trapping sites). Particularly reductive treatments not only provide SA surface mobility but also may affect the surface by creation of additional defects (e.g., oxygen vacancies in TiO_2_^[^ ^158^ ^]^) and thus potential trapping sites.

**Figure 9:**
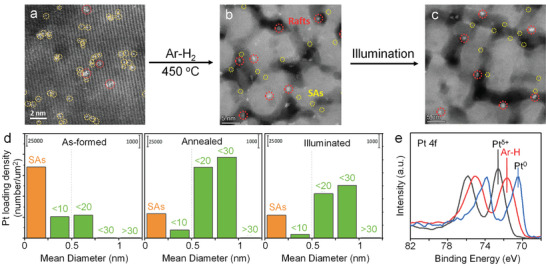
Schematic illustration of Ar–H_2_ treatment on TiO_2_ with Pt SAs. a) Pristine Pt SAs decorated TiO_2_, b) after Ar–H_2_ treatment, and c) after further light illumination. d) Particle size distribution of Pt in the different samples, and e) Pt 4f XPS spectra of different samples. Reproduced with permission.^[^ ^100^ ^]^ Copyright 2024, Wiley‐VCH.

In particular, annealing in an Ar–H_2_ atmosphere has been reported to be particularly beneficial for stabilizing SA configurations.^[^ ^100^ ^]^ **Figure** **9**a shows an example of a Pt SA decorated TiO_2_ surface that is subjected to annealing in an Ar–H_2_ atmosphere, resulting in mild agglomeration, forming 2D rafts ≈0.5–1 nm in diameter and consisting of 10–30 atoms (Figure 9b,d). These few‐atom‐agglomerates are particularly stable against further agglomeration, i.e., they remain stable even after being used in photocatalytic experiments (Figure 9c). The stability of such clusters indicates a strong interaction between few‐atom aggregates and the TiO_2_ surface. The stability can be attributed to the interaction of the few‐atom clusters with the defects of TiO_2_ formed under the reducing Ar–H_2_ atmosphere. Noteworthy is that these 2D rafts show a partially reduced state of Pt in XPS analysis (Figure 9e), i.e., represent a state between metal and oxygen‐coupled Pt SA.

#### 5.3.2 Stabilizing by Surface Functional Groups

Another stabilizing effect can be the placement of suitable neighbors to SAs. For example, on TiO_2_ there is a synergy effect of Pt SA with surface or surface‐near fluorides.^[^ ^147^, ^159^ ^]^ This is of a high relevance, as many TiO_2_ nanocrystalline powders or particularly preferentially facetted structures, like preferentially facetted (001) anatase nanosheets, are synthesized in HF environments and accordingly these structures the surfaces are to a large extent F‐terminated.^[^ ^160^, ^161^ ^]^

The terminal monolayer can be partially or fully removed by a treatment in hot water or sodium hydroxide (i.e., depending on immersion time, increasingly defluorinated surfaces can be produced).^[^ ^105^, ^147^ ^]^ Such surfaces show an enhanced activity and stability against Pt agglomeration^[^ ^147^ ^]^—effects that can be explained by the strongly electronegative nature of F that resonance‐stabilizes negative charge on the Pt (after photoelectron‐transfer to Pt). Other efforts involve the termination of surfaces with suitable coupling agents. For example, modifying a TiO_2_ surface with a silane monolayer has been shown to significantly enhance the efficiency of Pt SAs.^[^ ^162^ ^]^ Other work has reported that the hydroxyl group on anatase TiO_2_ can stabilize Ni single atoms by forming Ni_1_(OH)_2_ complexes.^[^ ^163^ ^]^

#### 5.3.3 3D Atomic Cavities

**Figure 10:**
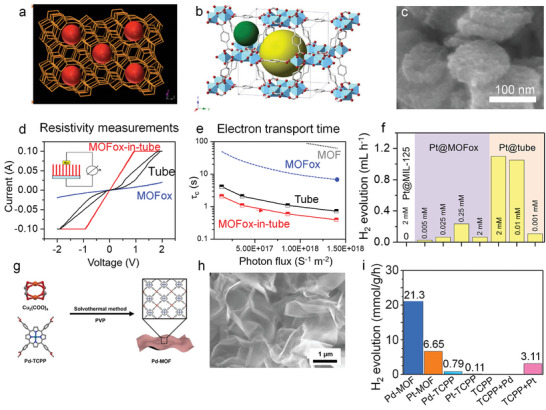
Schematic illustration of SAs embedded in a) zeolite, b) MOF, and c) SEM image of Pt SAs/MOF structure. d) Current–voltage curves for MOFox, TiO_2_ nanotubes, and MOFox‐in‐tube structure. e) Transport time constant values obtained from IMPS plots for MOFox powder, TiO_2_ nanotubes, and MOFox‐in‐tube structure. f) Comparison of photocatalytic H_2_ production performance among Pt decorated MOFox samples and TiO_2_ nanotubes, Reproduced with permission.^[^ ^170^ ^]^ Copyright 2023, Royal Society of Chemistry. g) Schematic illustration of the synthesis of Pd‐MOF, h) SEM image of Pd‐MOF nanosheets, and i) photocatalytic H_2_ production rate of different samples under visible light irradiation (*λ* = 450 nm, 500 mW cm^−2^). Reproduced with permission.^[^ ^171^ ^]^ Copyright 2024, Wiley‐VCH.

A classic approach to prevent SA agglomeration are 3D networks with suitable atomic‐size cavities, such as zeolite or MOF structures (**Figure** **10**a,b).^[^ ^118^, ^164^, ^165^ ^]^ An example based on a titania network is MIL‐125 (Figure 10b,c), which has a porous structure with uniform nanocavities <1 nm in diameter. This size is well able to accommodate single atoms, preventing them from migrating and clustering. Zeolite and MOF structures typically provide cavities large enough for embedding various size atoms and many frameworks are adjustable in size. Another advantage is the very high surface area that allows for high metal loading (if needed). In classic catalysis, namely, zeolite systems are widely used and embedded single atoms are usually very stable against agglomeration. However, for photocatalysis or electrocatalysis the main drawback of MOF or zeolite structures is their typically poor electrical or photoelectrochemical properties: typically, they show low electron mobility and a high recombination rate for excited carriers, as illustrated in Figure 10d,e.

Note that in Figure 10c pure Ti‐MOF structure (MIL‐125) is virtually an insulator,^[^ ^166^ ^]^ and even after modification of the MOFs with functional groups, such as ─NH_2_,^[^ ^167^, ^168^ ^]^ it remains insufficient to achieve the performance of traditional semiconductors like TiO_2_.

In literature this has often been “ameliorated” by heat treating the MOF (essentially converting the MOF to TiO_2_) but even these structures usually have still poor electrical properties (Figure 10d,e)—except for the fact that they are no longer MOF but essentially nanoporous TiO_2_ with still very short electron diffusion length.^[^ ^169^ ^]^

To address this problem, recently hybrid/hierarchical structures between high electric quality structures (TiO_2_ nanotubes) and MOF‐based porous oxides were developed for achieving a combination of electron transport and stabilization of SAs.^[^ ^170^ ^]^ This hybrid structure outperforms its constituents in electronic properties as well as in photocatalytic H_2_ production (Figure 10d,f), and particularly in its stability against light induced agglomeration.

#### 5.3.4 Coordination by Ligand within 2D‐MOF Structures

An even higher level of trapping SAs is achieved by making them a part of a molecular structure. There are many examples of this in homogeneous photocatalysis, particularly using metal organic complexes, as discussed in reviews on SAs in MOFs and related systems.^[^ ^6^, ^7^, ^119^, ^172^, ^173^ ^]^ Specific cases are 2D MOFs that are typically heterogeneous photocatalysts (not water soluble). The key advantage of the 2D geometry (in comparison with 3D MOFs) is that it provides more accessible active sites for chemical reactions and to light. Moreover, some 2D configurations enable efficient charge separation and transfer. Particularly various linked porphyrin structures have been highly efficient for (visible) light harvesting.^[^ ^174^, ^175^ ^]^ In this context, 2D MOFs with coordinated Pt or Pd single‐atom sites have been successfully prepared (Figure 10g).^[^ ^74^, ^171^ ^]^ Remarkably, the Pd‐MOF catalyst exhibits photocatalytic activity that is three times higher than that of Pt‐MOF and drastically higher than 2D non‐coordinated noble metal decorated TCPP (Figure 10h).^[^ ^171^ ^]^ This enhanced activity of Pd‐TCPP is contrary to general electrocatalytic models, which typically consider Pt superior to Pd for HER. The higher reactivity of Pd‐TCPP can in this case be attributed to a longer lifetime of the excited state within the Pd MOF. Such 2D MOFs with embedded suitable noble metal centers represent a promising path toward visible‐light‐active water‐splitting photocatalysts, as this approach offers a versatile toolbox for further optimization and innovation in the field of organometallic photocatalysis.

### 5.4 SAs in 3D Photoabsorbers: Optimizing Light and Charge Carrier Management

**Figure 11:**
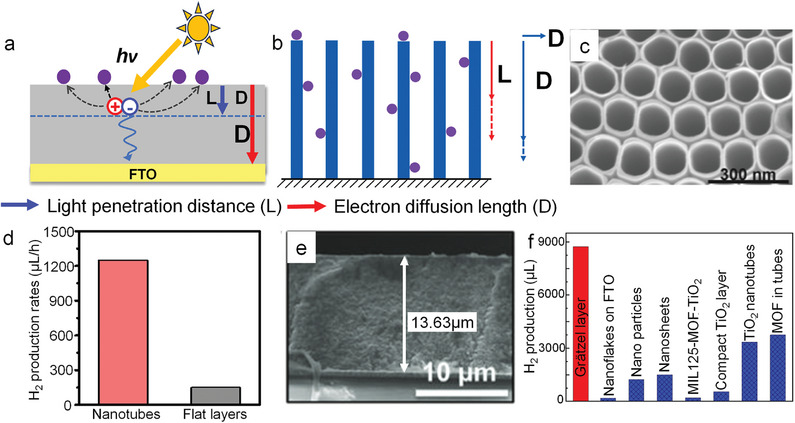
a,b) Schematic illustration of light penetration and electron diffusion in (a) flat layer and (b) 3D nanotube structure, and c) SEM image of TiO_2_. Reproduced with permission.^[^ ^176^ ^]^ Copyright 2007, Wiley‐VCH. d) Comparison of photocatalytic H_2_ production by TiO_2_ nanotubes and flat layers. e) Cross‐sectional SEM images of the Pt SAs decorated TiO_2_ layers. f) Comparison of photocatalytic H_2_ production by various TiO_2_ nanostructures with optimized Pt SA loading. Reproduced with permission.^[^ ^15^ ^]^ Copyright 2024, Wiley‐VCH.

Previous sections show that in many cases of photocatalytic use, already at very low SA loading density, not the cocatalyst but light harvesting becomes the rate determining issue.^[^ ^72^ ^]^ In order to improve light harvesting in semiconductor electrochemistry, often 3D (1D) structures such as nanotubes, ‐wires, and ‐rods are used to overcome the limitations of planar structures. In planar absorbers the efficiency of photocatalysis is limited by the mismatch between the light penetration distance (L) and the electron diffusion length (D) as illustrated in **Figure** **11**a.

In both photocatalysis and photoelectrodes a 3D architecture can improve the efficiency in light harvesting while optimizing charge carrier management. Using TiO_2_ as an example, a most popular 3D structure are TiO_2_ nanotubes (Figure 11c) that possess high electrical conductivity, which facilitates efficient charge transport and allow for orthogonal charge separation, i.e., the structure increases the light penetration distance, and the thin tube walls shorten the hole diffusion length (Figure 11b). Moreover, the nanotubes provide a large surface area and effective light scattering. These features contribute to a significantly improved light‐converting structure for hydrogen production.

The photocatalytic H_2_ production activity of Pt‐decorated nanotubes is over six times higher than that of Pt SAs decorated on flat TiO_2_ compact layers (Figure 11d). This significant increase in activity highlights the beneficial effect of the nanotube structure in enhancing photocatalytic performance.

Notably, even higher photocatalytic activity is achieved with Grätzel‐type nanoparticle layers.^[^ ^15^ ^]^ When these layers are sufficiently loaded with Pt SAs (Figure 11e), they exhibit significantly higher activity compared to other nanostructured TiO_2_ forms. In Figure 11f, the performance of various TiO_2_ structures including nanotubes, nanoflakes, nanoparticles, and nanosheets under the same conditions is compared. Clearly, the Grätzel‐type structure, combined with optimal platinum loading, provides a most effective configuration for photocatalytic hydrogen production among the tested nanostructures.

### 5.5 Characterization

Techniques commonly used to characterize single‐atom catalysts, either in classic catalysis or in photocatalysis are direct individual‐atom observation techniques, such as HAADF‐STEM or high‐resolution scanning tunneling microscopy (STM). Other frequently used techniques deliver averaged information, namely, on the chemical nature (oxidation state, nearest neighbors, steric influences) of a catalyst element, e.g., in a powdered sample, such as extended X‐ray absorption fine structure (EXAFS), X‐ray absorption near‐edge structure (XANES), diffuse reflectance infrared Fourier transform spectroscopy (DRIFTS), namely, CO‐DRIFTS, while XPS is the key tool for SAs on defined flat surfaces.

In this section, we will briefly introduce the common methods. **Table** **1** provides an overview of these methods, detailing the information and the target features of the techniques, and also points out common drawbacks and artifacts when addressing SAs with these techniques.

**Table 1:** Summary of different techniques for the characterizations of SAs.

Characterization techniques	Information	Advantages	Limitations
Microscopy techniques (mainly STEM)	Distribution, density, loading amount (EDX)	Direct imaging, high resolution	Interference of electron beam, Z‐contrast between metal‐supported atoms
XAS (including XANES and EXAFS)	Oxidation state, coordination number, symmetry, atom distance	Short‐range structural information(up to 5 Å), both surface and bulk phase	Not easily accessed, complex analysis process
XPS	Oxidation state, binding energy and surface composition	Surface sensitive	Only surface species and concentrations, low loading detection
FTIR (mainly DRIFTS)	Coordination state, functional groups	Distinguish between clusters and single atoms	Materials that react with CO or NO
NMR/EPR	Neighboring and spin states	High resolution, in situ	Only works in specific cases

Considering the variety of semiconductor substrates, numerous formats exist, including single crystals, powders, thin films, and complex 3D structures. Increasing complexity of these substrates adds challenges to the characterization of SAs on them. For example, a technique that is effective for single crystal surfaces or thin film may not be suitable for powders or 3D structures.

Moreover, not all SAs are equal (SAs at “hot” spots vs SAs at “dead” spots)—this is a common concern about the significance of averaging characterization methods. For instance, if a sample contains both SAs and NPs or agglomerates, averaging techniques may skew the evaluation toward the majority species, potentially overlooking the effects of the minority (but possibly highly reactive) species. Therefore, techniques that are able to characterize single sites in terms of chemistry, structure, and reactivity would be certainly most desirable—but in general, steps to this understanding require often idealized model systems.

Often there are “practical” restrictions to techniques, such as sample thickness for TEM, where samples cannot be too thick, as this would prevent the electron beam from effectively penetrating the sample, or limit the resolution and accuracy of the imaging. In this context it is also important to note that semiconductor substrates made from light elements, such as C_3_N_4_ and TiO_2_, benefit from a high Z‐contrast, which is particularly helpful for distinguishing between high Z single atoms and the low Z substrate.

#### 5.5.1 Microscopy Techniques

##### TEM

**Figure 12:**
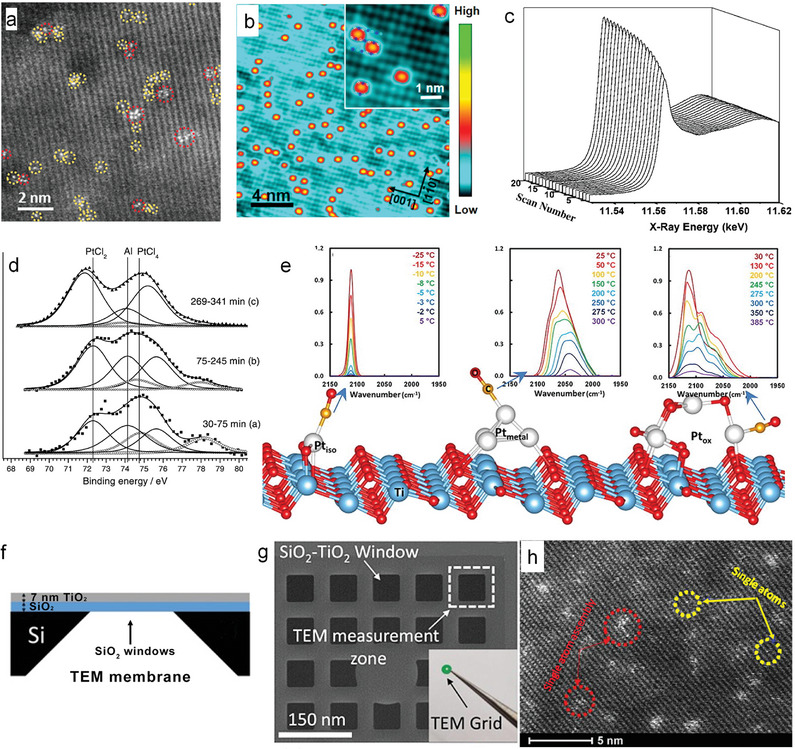
a) HAADF‐STEM images of the Pt SAs on TiO_2_ surface. Reproduced with permission.^[^ ^100^ ^]^ Copyright 2024, Wiley‐VCH. b) STM images of the Au SAs on CuO surface. Reproduced with permission.^[^ ^180^ ^]^ Copyright 2018, American Chemical Society. c) The XANES spectra for 1% Pt on silica. Reproduced with permission.^[^ ^127^ ^]^ Copyright 2004, Elsevier. d) Pt 4f XPS spectra of (5.0)Pt‐SF‐Cl catalyst during prolonged X‐ray bombardment. Reproduced with permission.^[^ ^181^ ^]^ Copyright 2003, Elsevier. e) CO TPD‐IR spectra from Pt_iso_, Pt_metal_, and Pt_ox_ catalysts and the corresponding structural model. Reproduced with permission.^[^ ^102^ ^]^ Copyright 2018, Elsevier. f) Schematic of the sputtered layer, g) top‐surface SEM image and (inset) optical image of the TiO_2_ layer deposited on SiO_2_‐Si TEM grid, and h) HAADF‐TEM image of Pt SA‐decorated TiO_2_ layer. Reproduced with permission.^[^ ^98^ ^]^ Copyright 2020, Wiley‐VCH.

The most widely used technique in the SA field is electron microscopy technique such as TEM and SEM. In view of SA characterization, advances in TEM techniques, particularly HAADF‐STEM, provide most compelling evidence of SA formation and distribution. HAADF‐STEM has rapidly passed STM as a most effective technique for identifying SAs. When coupled with EDS techniques, it allows the assessment of the dispersion and spatial distribution of SAs on the support, together with a rough quantitative estimate of SA density within a limited range of the sample surface. **Figure** **12**a presents a typical HAADF‐STEM image of Pt SAs on an anatase TiO_2_ surface.^[^ ^100^ ^]^ The isolated single atoms, as well as dimers and clusters, are visible as bright spots, clearly distinguishable from the support material due to their contrast differences.

Due to the comparable ease of use and direct proof for the presence of SA, virtually every SA study since 2003^[^ ^8^ ^]^ provides visualization of SAs using HAADF‐STEM. However, there are inherent limitations for SA characterization. Primarily, its reliance on Z‐contrast places specific demands on both the support material and the metal atoms under investigation. For instance, substrate materials that contain heavy elements such as Sc, Cd, Bi, Zn, and Hg exhibit minimal Z‐contrast to the heavy catalytic elements Pt, Pd, Rh, Au, Ir, etc., making it often challenging to convincingly identify the placement of SAs. This often leads to ambiguous results. Note that also using line scan approaches to try to resolve these problems can be further complicated by other STEM artifacts.^[^ ^177^ ^]^

In addition, the intense electron beam used in HAADF‐STEM can potentially alter sensitive materials and induce atomic migration on the surface.^[^ ^178^ ^]^ For example, Suzuta et al.^[^ ^179^ ^]^ prepared freestanding monolayer graphene decorated with Pt and Au SAs. Using STEM, they observed the dynamic behavior of these SAs under the interaction with the e‐beam. Their observations also reveal that Au SAs exhibit greater mobility on graphene than Pt SAs under identical electron beam irradiation conditions.

##### STM

Figure 12b shows an example of the use of STM for Au SA characterization on CuO.^[^ ^180^ ^]^ Classic uses of STM for SA imaging date back to the work of IBM Zurich,^[^ ^182^ ^]^ early work of SA identification in photocatalysis are examples of Pt SAs on Cu_2_O surfaces,^[^ ^183^ ^]^ or Pt SAs on anatase TiO_2_ (101) surface.^[^ ^184^ ^]^ STM is in most cases tedious to image a surface with atomic resolution but it can additionally provide insight into surface electronic properties and the ability to observe surface processes in real time. The latter can be critical for studies of dynamic phenomena in catalytic reactions. However, the utility of STM is limited by the requirement for conductive and sophisticatedly well‐prepared surfaces, with the potential for sample preparation artefacts, and in most cases the need for UHV conditions, which often do not represent the conditions of practical catalysis. Additionally, one may experience tip artifacts due to tip shape, contamination, or tip–sample interactions, etc.^[^ ^185^ ^]^ Due to these limitations, STM is often not regarded as a general approach for SA characterization.

##### SEM

It needs to be pointed out that SEM, although unable to directly visualize SAs, is also included here as a straightforward and effective method for identifying the absence of nanoparticles (i.e., above the resolution limit of a modern FE‐SEM ≈0.5 nm), and it can be used to indirectly inferring the presence of SAs. For example, if the presence of Pt can be detected by other techniques but is not visible in a modern FE‐SEM, this points to the presence of Pt units smaller than 0.5–1 nm. In particular, the interplay of FE‐SEM and XPS (on flat surfaces) allows very efficiently to scan large surfaces for the extent of NP versus SA contributions of a catalyst. This is of a considerable advantage for rapid parameter screening experiments.

#### 5.5.2 XAS/XANES/EXAFS

Several synchrotron techniques are based on X‐ray absorption spectroscopy (XAS), i.e., they measure the X‐ray absorption edge of an element. This includes XANES spectroscopy in the energy range 30 to 50 eV from the absorption edge and EXAFS spectroscopy from 50 to 1000 eV or beyond. The techniques are very useful for elucidating both the charge and near field geometry of materials or atoms. In characterizing SAs, the technique is often used to elucidate atoms oxidation state and next neighbor coordination.

Classic noble metal SA catalysts—foremost Pt SAs have been widely investigated by XANES to determine the oxidation state of Pt SAs, usually using PtO_2_ and a metallic Pt foil samples as reference to determine the oxidation state from the white line peak position.^[^ ^9^, ^17^, ^186^ ^]^ Also other metal SAs have been characterized by XANES, for example, Pd,^[^ ^187^, ^188^ ^]^ Ru,^[^ ^189^ ^]^ Ir,^[^ ^73^ ^]^ Rh,^[^ ^190^ ^]^ Ni.^[^ ^191^ ^]^

A modified evaluation of the X‐ray absorption edge is EXAFS, also used to determine the chemical states of elements (even at comparably low abundance or concentration). The EXAFS technique studies the fine structures in absorption at energies greater than the threshold for electron release, which provides information of the coordination (next‐neighbor atomic distance). Therefore, this technique is often regarded as a “must have” when characterizing metal SAs and investigating their coordination with the substrate, often also using comparison with reference peaks such as PtO_2_ and Pt.^[^ ^9^, ^188^, ^192^ ^]^

An example may be the early work by Qiao et al.^[^ ^9^ ^]^ who reported the synthesis of Pt SAs on FeO*_x_* via coprecipitation, with two different loadings: 0.17 wt% Pt (sample A) and 2.5 wt% Pt (sample B). XAS investigations indicated that the Pt atoms are positively charged, as evidenced by the features of white line intensity. The absence of second‐shell Pt–Pt coordination at a distance of 2.81 Å and the presence of Pt–Fe coordination at 2.88 Å provide direct evidence of the isolated nature of the Pt atoms in sample A. The first‐shell Pt–O coordination number at 1.9 Å suggests that the platinum atoms are likely stabilized on the FeO*_x_* surface through Pt─O─Fe bonds.

In spite of the wide use in literature, there are some points of consideration. First, the high X‐ray flux of synchrotron light sources can potentially cause “beam damage,” i.e., cause unwanted interactions of the X‐rays with the sample that alter its properties. This issue is particularly severe when detecting low‐concentration species or observing structural changes under in situ/operando conditions, where high detection sensitivity is needed, i.e., where a high photon flux is required. X‐ray‐induced changes also but need to be considered in studies of heterogeneous catalysts.^[^ ^193^, ^194^, ^195^ ^]^ For example, Miller et al.^[^ ^127^ ^]^ measured XANES spectra of 1% Pt on silica and observed a rapid increase in the white line region with subsequent scans. Initially, the sample contained Pt^2+^ but after ≈5.5 min, ≈50% of the Pt had been oxidized to Pt^4+^, and after 30 min, over 90% of the Pt was oxidized (Figure 12c). Albrahim et al.^[^ ^193^ ^]^ found that in a Rh/Al_2_O_3_ catalyst with highly dispersed Rh, higher X‐ray flux density can cause the reduction of Rh at room temperature and significant Rh agglomeration during the in situ reduction process, occurring within just 6 min of beam exposure. Therefore, also in SA characterization, control experiments may need to be considered to avoid misinterpretation of data and oxidation states determined by XANES/EXAFS considered with sufficient care.

Another point that should be noted is that the structure of metal SAs species is not directly determined via EXAFS. Instead, the measured data are fitted to a structural model, which depends largely on subjective interpretations regarding the statistical validity and differentiation from other structures. Additionally, EXAFS is an averaging technique, and the parameters derived from fitting represent the average of all different types of that element present in the sample.

#### 5.5.3 XPS

XPS is a widely used characterization technique that can probe the chemical and compositional properties of solid surfaces. It is particularly valuable for identifying SAs on compact (flat) samples as its information depth is in the 1 nm range. For example, Pt SAs, which typically have oxidation states ranging from 0 to +4, can be distinguished from metallic Pt (0) by their different binding energies. However, XPS is limited to the detection of surface species only and may have difficulty in providing information when the loading of SAs is low. Additionally, like other X‐ray‐dependent techniques, XPS can induce material transformations due to X‐ray beam exposure. For example, prolonged X‐ray exposure can reduce Pt(IV) to Pt(II).^[^ ^196^ ^]^ Karhu et al.^[^ ^181^ ^]^ studied the reduction process of Pt species on SiO_2_ supported Pt catalysts during XPS measurements, showing that after 45 min, PtCl_2_^2−^ was observed, and after ≈300 min, very little Pt remained in the form of PtCl_4_^2−^ (Figure 12d). This finding shows that such XPS data need to be considered sufficiently carefully. Moreover, it is important to point out that in literature some issues regarding the interpretation of XPS data seem to be not unusual. In some studies, improper spectral fitting—such as incorrect binding energy calibration, peak symmetry, and spin–orbit coupling—can be observed that can significantly affect the conclusions drawn from the research. Nevertheless, the ease of obtaining data with XPS makes this technique a key characterization tool when analyzing SA‐loaded surfaces.

#### 5.5.4 IR (DRIFTS)

DRIFTS is an infrared spectroscopy technique that uses diffuse reflection to collect data.^[^ ^197^ ^]^ To characterize SACs, DRIFTS typically uses a probe molecule such as CO. It relies on the fact that the CO molecule binds (vibrates dominantly in different modes) when attached to different noble metal configurations—see example in Figure 12e. The presence of a sharp, narrow peak in DRIFTS spectra indicates that CO is linearly adsorbed at isolated metal atom sites. For CO on Pt, characteristic peaks observed between 1700 and 2300 cm^−1^ correspond to different vibrational or stretching modes of CO adsorbed on Pt sites. Different Pt coordination environments can shift the frequency of these vibrations, with higher coordination leading to higher frequencies. The example in Figure 12e, Thang et al.^[^ ^102^ ^]^ demonstrates how CO‐FTIR can distinguish between different Pt species on anatase TiO_2_. The FTIR analysis of CO adsorption on these species revealed distinct spectral features: a symmetric, narrow, and stable band at 2113 cm^−1^ corresponding to atomically dispersed Pt atoms; a broader set of bands at 2028–2079 cm^−1^ attributed to Pt metal particles; and bands in the range of 2105–2117 cm^−1^ associated with cationic Pt species in oxidized Pt nanoparticles., i.e., isolated Pt atoms, Pt nanoparticles, and oxidized Pt nanoparticles are clearly distinguished. Similar investigations have been reported using CO‐FTIR to distinguish Pt SAs and Pt NPs on TiO_2_.^[^ ^186^ ^]^

It needs to be noted that the interpretation of these peaks can be context dependent. Factors such as the support material for the single metal atoms and their location—different coordination states or whether on the surface or embedded in the framework—can alter the CO adsorption peaks. Besides, because DRIFTS depends on the interaction of CO with surface adsorption sites, it cannot detect single atom sites that do not bind CO or SAs embedded in a bulk phase. Despite these few limitations, DRIFTS is a highly relevant tool in SA characterization.

#### 5.5.5 NMR/EPR

Nuclear magnetic resonance (NMR) and EPR are spectroscopic techniques that can probe the electronic and structural properties of materials. NMR techniques, particularly solid‐state NMR, offer insights into the structure and dynamics surrounding an NMR active species (isotope). This can be used to characterize the environment of catalytic centers in SACs, namely to identify the coordination environment of metal SAs and the binding sites. The choice of isotope for NMR analysis is often dictated by the natural abundance of the NMR active isotope and the sensitivity of the element under investigation. In the case of Pt, ^195^Pt is the only NMR‐active isotope, with a chemical shift that is readily observable and highly sensitive to changes in the oxidation state, ligand substitution, and the stereochemistry around the ^195^Pt nucleus. However, the low sensitivity of ^195^Pt NMR, coupled with the typically low surface concentration of metal sites (often less than 4 wt%), presents challenges for directly characterizing the local environment of Pt centers on surfaces using solid‐state NMR. Therefore, many NMR studies focus on investigating the anchoring sites of different metal SAs on the substrate, such as Rh,^[^ ^198^ ^]^ Pt,^[^ ^199^ ^]^ Au,^[^ ^200^ ^]^ Mo,^[^ ^201^ ^]^ etc. For example, Al NMR has been used to study the anchoring of catalytically active Pt on the surface of γ‐Al_2_O_3_.^[^ ^199^ ^]^

Recent efforts have focused on enhancing the sensitivity of conventional solid‐state NMR techniques to enable the characterization of Pt at low loadings.^[^ ^154^ ^]^ For instance, isolated Pt species have been successfully characterized by ^195^Pt NMR with a Pt loading of 3.7 wt% in a Pt complex precursor. However, in photocatalysis, where semiconductor‐supported Pt SAs typically have loadings below 1 wt%, detection remains challenging.

EPR is highly sensitive to unpaired electrons and provides information about the electronic environment and symmetry around an electronic spin active site, i.e., catalytic sites and the fate of electrons in redox reactions. For example, EPR spectroscopy can often effectively detect metal ions in different oxidation states by observing characteristic hyperfine splitting patterns in the spectra. Typically, a comparative analysis is performed between the EPR spectra of the support material and the support decorated with SAs. The changes in the spectra caused by the introduction of SAs provide valuable insights into the interactions between the SAs and the support material. This comparative approach helps to understand the electronic environment and the extent of the interaction, which are crucial for optimizing the catalytic performance of the SAs.

The technique known for its high sensitivity and high resolution is very useful in the mechanistic study of SAs. However, the application can be limited to specific cases where the materials must exhibit sufficient magnetic response. They are often most effective when combined with other characterization techniques.

#### 5.5.6 In Situ Techniques

With the rapid advancement of characterization methods, in principle in situ techniques are an essential tool for investigating SACs. Some methods can offer valuable insights into the dynamic behavior and structural evolution of SACs under realistic operating conditions, enabling direct or indirect observation of changes in electronic structure, atomic dispersion, coordination environment, and chemical states in real time during photocatalytic reactions. Techniques such as STM, HRTEM, XAS, and CO‐FTIR have been used in this context, as comprehensively summarized in several review articles.^[^ ^202^, ^203^, ^204^ ^]^

However, it is important to acknowledge that in situ techniques, namely, using focused electrons or high‐energy X‐rays, do not always reflect real catalytic and dynamic reactions. Some of the characterization methods can strongly alter sample properties, as illustrated in Figure 12c,d. Additionally, the reliability of these results often depends on the stability of the support material; for example, highly stable supports are crucial for minimizing damage from electron beams. In cases of pseudo in situ measurements, using well‐defined model systems is necessary to achieve more accurate and meaningful characterization, as discussed below.

##### Thin Film Models

To provide an experimental photocatalyst model system that allows a maximum of well‐defined characterization of SAs as well as the reactivity, thin film model systems have recently been introduced as shown in Figure 12f–h.^[^ ^98^ ^]^ This setup can often serve as a substitute for XAS methods on well‐defined compact thin‐film substrates. The experimental platform generally consists of thin sputter‐deposited layers on a photolithographically defined TEM window grid. The photoabsorber layers (here TiO_2_) are in the range of 10–50 nm thick, sputter‐deposited on a 5–10 nm thick SiO_2_ window. This structure is highly TEM transparent (providing for atomic resolution for site‐specific detection of positions) and allowing the extraction of extensive data on SAs by combining HAADF‐STEM, FE‐SEM, and XPS techniques, as well as photocatalytic performance characterization all on one sample.

The design also allows for repeated tracing of atomic positions, even after multiple reaction steps or prolonged reaction times. This makes the platform an ideal choice for pseudo in situ observations.

## 6 Applications

SAs have been widely explored in various directions of photocatalytic applications. While the primary focus has been on photocatalytic water splitting (H_2_ generation), other applications such as CO_2_ reduction, photocatalytic N_2_ fixation, pollutant degradation, and organic synthesis (upconversion) are increasingly explored. In this case, noble metal SAs are used (and investigated) simply following concepts established prior with noble metal nanoparticles as cocatalysts.^[^ ^205^, ^206^ ^]^ In these applications, noble metal catalyst SAs are primarily used to maximize utilization of metal atoms and they serve for mediating electron (or hole) transfer. Much less, noble metal SAs have been found to alter the reaction mechanisms, i.e., not only to affect performance but also the speciation of reaction products or reaction pathway.^[^ ^14^ ^]^

The following sections summarize the use of SACs in various photocatalytic applications, highlighting their development and challenges.

### 6.1 Photocatalytic Water Splitting

As outlined in previous sections, heterogeneous single‐atom aided photocatalysts have been widely examined for photocatalytic HER. The most investigated systems are Pt/TiO_2_ and Pt/C_3_N_4_ SAC. Both SA systems demonstrate superior hydrogen production efficiency compared to nanoparticle‐based catalysts. Following the pioneering work by Yang and colleagues,^[^ ^18^ ^]^ numerous studies have been carried out on the deposition of SAs on semiconductors for photocatalytic H_2_ production.

Several experimental studies demonstrate that SACs with single Pt atoms achieve not only a higher mass‐specific H_2_ evolution, but also higher hydrogen evolution rates in terms of site‐specific activity, with TOFs substantially higher than their nanoparticle counterparts. This is attributed to the highly efficient electron transfer between the single metal atoms and the support, enhancing the separation of charge carriers and increasing the overall photocatalytic performance and the fact that nanoparticles—in contrast to SAs—may also catalyze the backward reaction.^[^ ^207^ ^]^ In addition to Pt, other metals, such as Cu and Co, have been decorated on TiO_2_ for catalyzing the HER. For example, Cu SAs on TiO_2_ were reported to show reversible and cooperative photoactivation.^[^ ^65^ ^]^ During the photocatalytic process, the Cu SAs undergo a valence state change due to the atomistic localization of photogenerated electrons. This, in turn, activates TiO_2_ to a more catalytically active state, with the process being reversible, returning to the initial state after the reaction.

In general, even more studied than TiO_2_ as a support for SAs is C_3_N_4_, due to its well‐defined coordination sites for Pt SAs and a full range of other transition metals. C_3_N_4_ has a nitrogen‐rich structure with abundant lone pairs of electrons, which can create strong metal–N coordination sites. These sites can effectively anchor metal SAs, preventing their aggregation and ensuring a high dispersion of active sites, which enables precise control over cocatalyst distribution and activity. So far numerous metal SAs have been decorated on C_3_N_4_ for photocatalytic H_2_ production, including Pt,^[^ ^62^, ^106^, ^208^, ^209^ ^]^ Pd,^[^ ^210^, ^211^, ^212^ ^]^ Au,^[^ ^213^ ^]^ Ag,^[^ ^214^ ^]^ Rh,^[^ ^211^ ^]^ Ru,^[^ ^211^, ^215^ ^]^ Cu,^[^ ^216^ ^]^ Fe,^[^ ^217^ ^]^ Co,^[^ ^218^ ^]^ Ni,^[^ ^219^ ^]^ etc.

For TiO_2_, the most commonly studied polymorphs include anatase, rutile, and mixed‐phase P25 nanoparticles. The choice of polymorph often depends on the specific photocatalytic application. For example, the anatase phase is predominantly investigated for photocatalytic H_2_ production, while other polymorphs are more frequently used for photodegradation processes.^[^ ^29^ ^]^ A comparative study examined the efficiency of Pt SAs on different TiO_2_ polymorphs and found that, under similar Pt loading, P25 exhibited the highest H_2_ production efficiency, followed by anatase, with rutile showing the lowest performance.^[^ ^151^ ^]^

Particularly noteworthy are preferentially faceted single‐crystalline sheets, which can enhance photocatalytic activity due to their well‐defined surface facets that promote specific reactions. Research has focused on controlling the faceting of TiO_2_, mainly anatase TiO_2_, to achieve dominant facets that can facilitate charge transfer and accumulation for targeted reactions. For instance, electrons are known to migrate to the (101) facets while holes accumulate at the (001) facets. Consequently, a cocatalyst for H_2_ evolution is conceptually only required on the (101) facets. Building on this concept, a recent study selectively deposited Pt SAs on the minority (101) facets and found that only a minimal amount (0.005 wt%) of Pt SAs was necessary to achieve maximum H_2_ production.^[^ ^105^ ^]^

Although Pt is widely recognized for its high efficiency as a cocatalyst for HER, other transition metals have also been explored due to their lower cost. For example, Co, Cu, and Ni SAs have been decorated on TiO_2_ to enhance H_2_ production by improving electron conductivity and charge separation,^[^ ^65^, ^220^ ^]^ albeit in most cases (exceptions discussed later) not as effective as Pt. An even more promising approach involves using transition metal SAs on C_3_N_4_, where the Co–N_4_ structure is perceived to mimic the active sites of biological enzymes,^[^ ^107^ ^]^ offering a cost‐effective alternative to Pt‐based systems by enhancing reaction kinetics. Cao et al.^[^ ^218^ ^]^ prepared Co SAs decorated C_3_N_4_ with Co–N_4_ coordination, and it demonstrates efficient photocatalytic H_2_ production performance. The Co–N_4_ geometry serves as an active center for facilitating H─H bond formation, thereby significantly improving H_2_ evolution efficiency.

**Figure 13:**
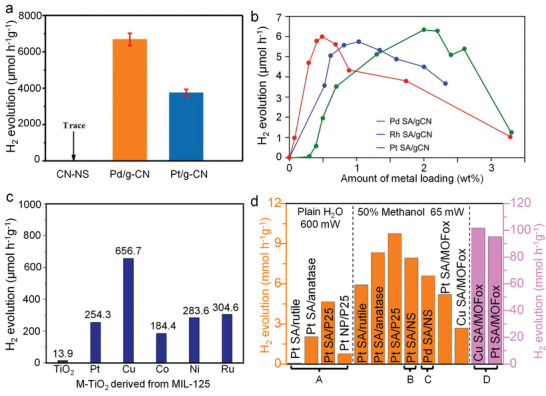
a) Comparison of photocatalytic H_2_ production performance of C_3_N_4_ and C_3_N_4_ decorated with Pd or Pt SAs. Reproduced with permission.^[^ ^210^ ^]^ Copyright 2018, Wiley‐VCH. b) Comparison of photocatalytic H_2_ production performance of C_3_N_4_ and C_3_N_4_ decorated with Pd, Pt or Rh SAs. Reproduced with permission.^[^ ^212^ ^]^ Copyright 2023, Wiley‐VCH. c) The mass‐specific H_2_ generation rates achieved on TiO_2_ derived from a metal–organic framework and loaded with various metals. Reproduced with permission.^[^ ^222^ ^]^ Copyright 2023, Springer Nature. d) Comparison of photocatalytic H_2_ production performance of different metal SAs loading on different TiO_2_ polymorphs. Data adapted from A,^[^ ^151^ ^]^ B,^[^ ^152^ ^]^ C,^[^ ^221^ ^]^ and D.^[^ ^224^ ^]^

In view of seeking the best cocatalyst, many investigations compare the efficiency of different metal SAs cocatalyst. For example, TiO_2_ was decorated with Pt, Pd, and Au SAs, and interestingly, Pd SAs demonstrated the highest cocatalytic activity.^[^ ^221^ ^]^ This was attributed to more efficient charge localization on Pd SAs. Similar investigations have been conducted on the C_3_N_4_ system, where Pd SAs decorated on C_3_N_4_ demonstrated a higher H_2_ production rate compared to Pt SAs on C_3_N_4_ (**Figure** **13**a).^[^ ^210^ ^]^ However, the comparison is not entirely rigorous, as the Pd loading was 0.13 wt%, while the Pt loading was 0.96 wt%, resulting in TOFs of 417 h^−1^ for Pd SAs and 76 h^−1^ for Pt SAs. A more comprehensive study by Akinaga et al. compared Pt, Pd, and Rh SAs.^[^ ^212^ ^]^ As shown in Figure 13b, they found that different metal SAs achieve maximum H_2_ production efficiency at different loadings, such as ≈0.5 wt% for Pd, ≈1 wt% for Rh, and ≈2 wt% for Pt. Consequently, Pd SAs were identified as the most effective cocatalyst for H_2_ production. These results differ from recent findings, where maximum H_2_ production efficiency was achieved at a very low loading of 0.03 wt% for Pt SAs.^[^ ^77^ ^]^ Similar trends were also observed in MOF‐based single‐atom photocatalysts, where Pd‐MOFs exhibited significantly higher activity than Pt‐MOFs (Figure 10i). Wang et al.^[^ ^222^ ^]^ reported that Cu SAs on TiO_2_, derived from an MOF, outperformed Pt SAs in photocatalytic H_2_ production (Figure 13c). It remains unclear, though, whether this difference arises from the intrinsic activity of the metal SAs or from specific structural interactions between the metal and the MOF framework. Subsequent studies have not been able to reproduce these results (Figure 13d). These investigations compared the photocatalytic performance of Pt, Pd, and Cu SAs on different TiO_2_ polymorphs, as well as a comparative sample prepared using literature methods (Figure 13d), consistently showed that Cu SAs exhibited lower efficiency than Pt SAs on the same TiO_2_ substrate. These conflicting results do not necessarily indicate inaccuracies but highlight the challenges in directly comparing findings across different studies. One of the key problems being the noncomparable reactor designs (as widely outlined in classic photocatalytic literature^[^ ^223^ ^]^). These varying results suggest: first, under specific conditions other metal SAs may outperform Pt as cocatalysts; second, the interaction between the metal SAs and the substrate plays a critical role; and third, direct comparison of performance is almost only possible if identical set‐ups (not only light sources and intensity) are compared. While Pt SAs may be highly efficient on certain substrates, specific metal–substrate configurations can yield exceptions. Comparisons as in **Table** **2** have to be treated with utmost care, as key factor in the influence is the different reactor geometry in connection with the light intensity dependence, as discussed earlier (Figure 5b). One level more reliable are works that compare different systems with the same setup (as shown in Figure 13d).

**Table 2:** Summary of SACs for photocatalytic H_2_ production.

Catalysts with metal SA loading	Reaction conditions	Performance		Refs.
		Activity [µmol h^−1^ g^−1^]	Efficiency	
Pt/TiO_2_ (0.16 at%)	MeOH/H_2_O (v:v = 1:1) 365 nm LED, 65 mW cm^−2^	293 µmol h^−1^ cm^−2^	TOF: 2844 h^−1^	[15]
Pt/TiO_2_ (0.02 wt%)	MeOH/H_2_O (v:v = 1:4) 300 W Xe lamp	52 720	TOF: 51 423 h^−1^	[225]
Pd/TiO_2_ (0.16 at%)	MeOH/H_2_O (v:v = 1:3) 300 W Xe lamp	24 600	AQY: 23.4% (365 nm)	[226]
Cu/TiO_2_ (1.5 wt%)	MeOH/H_2_O (v:v = 2:1) 0.5 Sun (AM1.5G)	101 700	AQE: 56% (365 nm)	[224]
Ru/TiO_2_ (0.29 wt%)	Glycol/H_2_O (v:v = 1:9) 3.3 Sun (AM1.5G)	17 810	AQE: 21.3% (365 nm)	[227]
Ni/TiO_2_ (0.46 wt%)	MeOH/H_2_O (v:v = 1:9) 0.5 wt% Pt cocatalysts 300 W Xe lamp	1890	–	[228]
Ag/C_3_N_4_ (3.7 wt%)	TEOA/H_2_O (v:v = 1:9) 300 W Xe lamp, *λ* > 420 nm	1866	–	[214]
Pd/C_3_N_4_ (0.5 wt%)	MeOH/H_2_O (v:v = 1:9) 300 W Xe lamp, *λ* > 410 nm	57	–	[212]
Pt/C_3_N_4_ (2.32 wt%)	TEOA/H_2_O (v:v = 1:9) 300 W Xe lamp, *λ* > 420 nm, 100 mW cm^−2^	1125	AQE: 15.2% (450 nm)	[229]
Co/C_3_N_4_ (1.0 wt%)	TEOA/H_2_O (v:v = 2:8) AM1.5G	216	AQE: 3.02% (450 nm)	[218]
Fe/C_3_N_4_ (0.5 at%)	TEOA/H_2_O (v:v = 1:9) 3 wt% Pt cocatalysts 300 W Xe lamp, *λ* > 420 nm	3390	AQE: 6.89% (420 nm)	[217]
Ni/C_3_N_4_ (0.2 at%)	TEOA/H_2_O (v:v = 1:9) 300 W Xe lamp	354.9	–	[219]
Cu/C_3_N_4_ (0.85 wt%)	TEOA/H_2_O (v:v = 1:9) 3 wt% Pt cocatalysts, 300 W Xe lamp	3261	AQY: 7.1% (420 nm)	[216]
Pt/CdS (0.91 wt%)	TEOA/H_2_O (v:v = 1.5:8.5) pH = 9, 0.5 Sun (AM1.5G)	6350	AQE: 25.7% (500 nm)	[230]
Ni/ZnIn_2_S_4_ (0.9 wt%)	TEOA/H_2_O (v:v = 1:9) 300 W Xe lamp, *λ* > 420 nm	1788	AQE: 9.6 (420 nm)	[231]
Pt/MOF (12 wt%)	0.1 m ascobic acid 300 W Xe lamp, *λ* > 420 nm	11 320	–	[74]
Pt/COF (0.72 wt%)	Sodium ascorbate in PBS buffer (4 mg mL^−1^), *λ* > 420 nm, 265 mW cm^−2^	719	AQE: 0.38 (420 nm) TOF: 19.5 h^−1^	[232]
Pt/Cs_2_SnI_6_ (0.12 wt%)	H_3_PO_2_/HI (v:v = 2:8) *λ* > 420 nm, 100 mW cm^−2^	430	TOF: 70.6 h^−1^	[233]
Pt/FAPbBr_3_ (1.8 wt%)	H_3_PO_2_/HI (v:v = 5:2) AM1.5G	6826	STH: 4.5%	[234]

### 6.2 Photocatalytic CO_2_ Reduction

The photoreduction of CO_2_ to fuels and value‐added chemicals (such as CO, CH_4_, and C_2_ products) has emerged as a promising technique for converting CO_2_, a currently globally highly investigated greenhouse gas, to useful products.^[^ ^235^ ^]^ The primary goal in this field is to find pathways that can overcome the very sluggish kinetics and increase the conversion rate of CO_2_ molecules. In fact, CO_2_ conversion is much more “difficult” to efficiently achieve than H_2_ production. This is due to a number of factors, namely, the high thermodynamic stability of the linear CO_2_ molecule and the C═O bonds, and, multiple and complex reaction pathways that yield a diverse range of reduction products. A significant practical challenge in photocatalytic CO_2_ reduction is avoiding the dominance of the unwanted side reaction of water to H_2_ during the process.

Generally, photocatalytic CO_2_ reactions are explored in either gas phase or liquid phase reactors (liquid phase means CO_2_ absorbed in alkaline solutions or present directly as carbonate solutions).^[^ ^236^ ^]^

The reaction products differ depending on the phase: in the gas phase, typical products include CO, CH_4_, and C_2_H_6_, while in the liquid phase, HCOOH, CH_3_OH, and other oxidized hydrocarbons are more commonly observed.^[^ ^236^ ^]^ In general, the vast majority of reactions are carried out in the gas phase. To date, various metal SAs have been used for photoreduction of CO_2_, including Pt,^[^ ^17^ ^]^ Rh,^[^ ^190^ ^]^ Ru,^[^ ^237^ ^]^ Au,^[^ ^238^, ^239^ ^]^ Ag,^[^ ^16^ ^]^ Fe,^[^ ^240^ ^]^ Co,^[^ ^241^ ^]^ Ni,^[^ ^242^ ^]^ Cu,^[^ ^243^, ^244^, ^245^ ^]^ etc., on various semiconductors.

Most work on SA catalysts follows the classic work on CO_2_ photocatalytic reduction that generally found that Pt, Ru, Cu, etc., are useful cocatalysts for this reaction.^[^ ^246^, ^247^, ^248^ ^]^ One of the most researched cocatalysts for CO_2_ reduction are Pt‐based NPs and thus also SAs. However, the products for photocatalytic CO_2_ reduction over Pt NPs and Pt SAs are typically CO and CH_4_, and it is particularly challenging to directly produce C_2_ products using SACs.

When Pt is atomically dispersed on TiO_2_ or other semiconductors, the catalytic efficiency of CO_2_ reduction increases significantly. It is generally reported that isolated Pt atoms facilitate the adsorption and activation of CO_2_, enabling its conversion into value‐added products like CO or CH_4_, while Pt nanoparticles tend to favor the competing HER process.^[^ ^17^ ^]^

Furthermore, other metals like Cu and Fe are also frequently explored as SACs for CO_2_ reduction.^[^ ^240^, ^243^, ^244^, ^245^ ^]^ For example, Cu SACs on TiO_2_ have shown promising results in reducing CO_2_ to CO, ascribed to the strong interaction between Cu atoms and the CO_2_ molecules at the atomic scale.^[^ ^245^ ^]^

Another alternative to noble metals are Co atoms, dispersed on semiconductor supports like g‐C_3_N_4_,^[^ ^249^ ^]^ that enhance the adsorption of CO_2_ molecules and facilitate their reduction to typical products like CO and CH_4_. In analogy, also some electrocatalytic studies emphasize the importance of Co–N_4_ sites in mimicking natural enzymatic systems for efficient CO_2_ reduction.^[^ ^250^ ^]^

While classic semiconductive compounds such as TiO_2_ and CdS have been used increasingly,^[^ ^238^, ^244^, ^245^ ^]^ also other metal oxides and sulfides (V_2_O_5_,^[^ ^251^ ^]^ ZrO_2_,^[^ ^252^ ^]^ In_2_O_3_,^[^ ^190^, ^253^ ^]^ CeO_2_,^[^ ^237^ ^]^ ZnIn_2_S_4_,^[^ ^239^, ^241^ ^]^ etc.) have been explored. And more recently, the development of metal SAs on heterojunction structures, such as Cu SAs on TiO_2_/BiVO_4_,^[^ ^254^ ^]^ Fe SAs on TiO_2_/SrTiO_3_,^[^ ^255^ ^]^ has shown promising results for enhanced photocatalytic CO_2_ reduction efficiency. One study reported the photoreduction of CO_2_ using Ni SAs decorated on ZrO_2_.^[^ ^252^ ^]^ Nevertheless, given that ZrO_2_ is typically considered an insulator, a classic photocatalytic mechanism is somewhat questionable.

**Figure 14:**
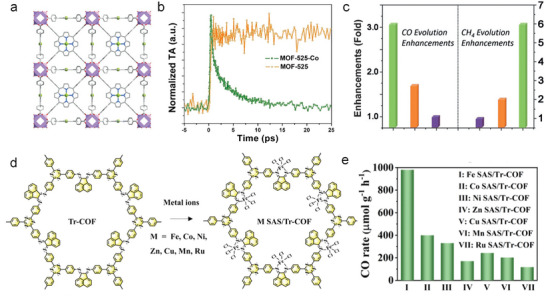
a) Structure model of MOF‐525‐Co, b) ultrafast transient absorption signal as a function of probe delay for MOF‐525 and MOF‐525‐Co (probed at 450 nm), and c) enhancement of production evolution over MOF‐525‐Co (green), MOF‐525‐Zn (orange), and MOF‐525‐Zn (purple). Reproduced with permission.^[^ ^258^ ^]^ Copyright 2016, Wiley‐VCH. d) Synthetic scheme and e) different metal SAs loaded in Tr‐COFs for CO_2_ photoreduction in 1 h reaction. Reproduced with permission.^[^ ^257^ ^]^ Copyright 2022, American Chemical Society.

Most research efforts on SAC‐based photocatalytic CO_2_ reduction has focused on materials such as C_3_N_4_,^[^ ^256^ ^]^ MOFs,^[^ ^243^ ^]^ and COFs.^[^ ^257^ ^]^ For example, Co SAs incorporated into a porphyrin‐based MOF structure have been used for the photoreduction of CO_2_ (**Figure** **14**a).^[^ ^258^ ^]^ The incorporation of Co SAs significantly improves the electron–hole separation efficiency in porphyrin units (Figure 14b), which greatly enhances the photocatalytic conversion of CO_2_ to CO and CH_4_—achieving a three‐ to sixfold increase compared to the bare MOF (Figure 14c).

Also in this context it should be mentioned that when using MOFs as photocatalysts, a common challenge is their ionic bonding nature, which typically results in large bandgaps and poor electrical conductivity (see Figure 10d,e).^[^ ^259^ ^]^ This leads to inefficient electron transport, limited photon‐to‐electron conversion, and poor visible light absorption. Although considerable efforts have been made to develop conductive MOFs that retain their intrinsic properties while enhancing electronic conductivity through d–π conjugation between organic ligands and metal nodes.^[^ ^260^ ^]^ However, electron mobilities for typical MOFs, e.g., MIL‐125, are ≈10^−5^ cm^2^ V^−1^ s^−1^.^[^ ^166^ ^]^ This is a stark contrast to semiconductors like TiO_2_, which exhibit electron mobilities of ≈1–10 cm^2^ V^−1^ s^−1^.^[^ ^261^ ^]^

Different metal SAs can significantly affect the kinetics of CO_2_ reduction and the selectivity of the products. For example, Ran et al.^[^ ^257^ ^]^ incorporated seven different metal SAs into a Tr‐COF structure (Figure 14d) and found that Fe SAs decorated COF exhibit the highest CO production rate (Figure 14e).

The selectivity of the product depends not only on the metal SAs but also on the support material. For example, Li et al.^[^ ^262^ ^]^ prepared a Cu SAs decorated g‐C_3_N_4_ photocatalyst that achieves ≈100% CO selectivity due to the CO_2_ adsorption site characteristics on the Cu SA. In contrast, other studies reported that Cu SAs on TiO_2_^[^ ^263^ ^]^ and WO_3_^[^ ^264^ ^]^ achieve selectivity of 83.3% for CH_4_ and 67% for CH_3_COOH, respectively. A study by Gao et al.^[^ ^61^ ^]^ used DFT calculations to compare the performance of Pt SAs and Pd SAs on C_3_N_4_ for CO_2_ photoreduction. The results suggest that HCOOH is the preferred product on Pd SAs decorated C_3_N_4_, with a rate‐determining barrier of 0.66 eV. In contrast, Pt SAs decorated C_3_N_4_ tend to reduce CO_2_ to CH_4_, with a rate‐determining barrier of 1.16 eV. Theoretical studies offer an efficient approach to evaluate reaction pathways and guiding the selection of specific SAs for desired products. However, real‐world reactions may differ due to factors such as the actual coordination state of the SAs, their loading, and distribution. **Table** **3** gives a summary of SACs for photocatalytic CO_2_ reduction, summarizing various metal single atoms, loadings, and supports, and highlighting their impact on photocatalytic CO_2_ reduction activity and selectivity.

**Table 3:** Summary of SACs for photocatalytic CO_2_ reduction.

Catalysts (SA loading)	Reaction conditions	^13^CO_2_ isotope labeling experiment	Performance		Refs.
			Activity [µmol h^−1^ g^−1^]	Selectivity	
Cu/TiO_2_	CH_3_CN/H_2_O (v:v = 5:1), CO_2_, 300 W Xe lamp	Yes	13.8	64.8% QE: 0.48% (385 nm)	[244]
Cu/TiO_2_ (0.17 wt%)	H_2_O, CO_2_ AM1.5G	Yes	88.34	CH_4_: ≈94%	[245]
Ag/TiO_2_ (0.23 wt%)	H_2_O, CO_2_ AM1.5G	Yes	46	CH_4_: 91%	[268]
Re/In_2_O_3_ (2 wt%)	H_2_O, CO_2_ 300 W Xe lamp	Yes	265.6	CH_3_OH: 100%	[190]
Ru/CeO_2_ (0.5 wt%)	H_2_O, CO_2_ 300 W Xe lamp	Yes	3.71	CH_4_: 92% AQY: 0.51% (450 nm)	[237]
Au/CdS (1.0 wt%)	H_2_O, CO_2_ 300 W Xe lamp, 600 mW cm^−2^	Yes	32.2	CO: 62%	[238]
Cu/WO_3_ (3 at%)	H_2_O, CO_2_ 300 W Xe lamp	No (in situ DRIFTS)	2.87	CH_3_COOH: 67%	[264]
Cu/TiO_2_/BiVO_4_ (0.13 wt%)	H_2_O, CO_2_ 300 W Xe lamp	Yes	17.33	CO: 100%	[254]
Au/ZnIn_2_S_4_ (0.24 wt%)	CH_3_CN/water, TEOA, Ru‐(bpy)_3_)Cl_2_·6H_2_O *λ* > 420 nm	Yes	275	CH_4_: 77% AQY: 0.12% (450 nm)	[239]
Pt/C_3_N_4_ (1.08 wt%)	H_2_O, CO_2_ AM1.5G	Yes	6.3	CH_4_: 99%	[17]
Ag/C_3_N_4_ (0.73 wt%)	H_2_O, CO_2_ AM1.5G	Yes	160	CO: >94% AQE: 4.8% (365 nm)	[16]
Ni/C_3_N_4_ (7.96 wt%)	H_2_O, CO_2_ 300 W Xe lamp	No (in situ DRIFTS)	8.6	CO: 81.1%	[242]
Cu/C_3_N_4_ (10.95 at%)	H_2_O, CO_2_ 300 W Xe lamp,	No (in situ DRIFTS)	3.086	CO: ≈100%	[262]
Co/MOF‐525	MeCN/TEOA = 4:1 400 nm < *λ* < 800 nm	Yes	200.6	CO: 84.5%	[258]
Fe/Tr‐COF	CH_3_CN/water, TEOA, Ru‐(bpy)_3_)Cl_2_·6H_2_O 300 W Xe lamp, *λ* > 420 nm	Yes	980.3	CO: 96.4%	[257]

A very important critical point is that in some studies there is uncertainty about whether the carbon products genuinely originate from CO_2_ or from other carbonaceous sources.^[^ ^265^, ^266^ ^]^ This is even more the case for studies that use organic semiconductors, light absorbers, or cocatalysts, and lack proper reference experiments, namely, using isotope labeling and control experiment. While the use of ^13^CO_2_ isotope labeling has become more common in recent studies, as illustrated by the examples in Table 3, the lack of standardized protocols remains a significant obstacle and can sometimes result in false‐positive outcomes.^[^ ^267^ ^]^ Furthermore, the field still lacks accurate and effective methods for isotope detection across various products in CO_2_ photoreduction, leading to skepticism within the reported results. These challenges are not unique to CO_2_ reduction but are especially critical in the context of single‐atom photocatalysts for CO_2_ reduction.

### 6.3 Photocatalytic N_2_ Fixation

N_2_ fixation, i.e., the reaction of N_2_ to NH_3_, typically requires high temperatures and pressures in current industrial settings (e.g., the Haber–Bosch process). In principle photocatalysis may provide a sustainable route for nitrogen fixation. Again, cocatalysts may provide active sites that facilitate the reduction of N_2_ to ammonia (NH_3_) under ambient conditions (or alternatively, the oxidation of N_2_ to nitrates).

However, progress in this area has been limited, with only comparably little success. The main challenge for N_2_ fixation is the activation and cleavage of the strong, nonpolar N≡N triple bond with a bond energy of 941 kJ mol^−1^. This challenge is particularly pronounced under the conditions of photocatalysis, as photogenerated electrons tend to recombine with their holes rather than to activate the N≡N bond. As with CO_2_ reduction, a significant challenge is avoiding the dominance of the unwanted side reaction of HER during the process.

A classic systematic study by Ranjit et al.^[^ ^269^ ^]^ on noble metal‐loaded TiO_2_ demonstrated that certain noble metal cocatalysts can provide an enhanced NH_3_ formation. The observed order of photoactivity was Ru > Rh > Pd > Pt, which correlated with the strength of the metal–H bond. This and other related work suggest that noble metals with a higher activation barrier for H_2_ evolution can lead to increased NH_3_ yields. Nevertheless, NH_3_ production rates remain typically low (close to background contamination), and thus activity in this field remained relatively low.

**Figure 15:**
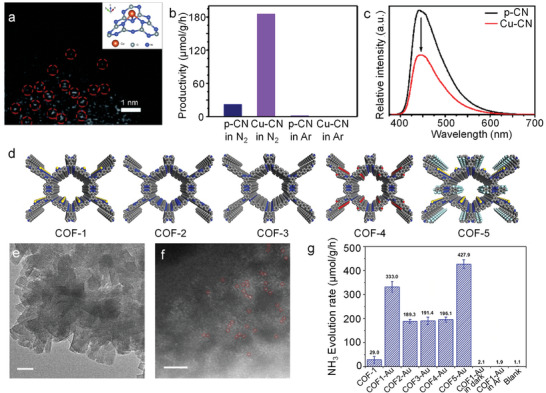
a) HAADF‐STEM and structure model of Cu SAs/C_3_N_4_. b) NH_3_ evolution rates of different catalysts in different conditions and c) PL spectra. d) Top views of COFX, e) TEM, and f) HAADF‐STEM image of Au SAs/COF1, and g) NH_3_ evolution rates of different catalysts in different conditions.

The use of metal SAs in N_2_ fixation began to gain momentum in 2018, with most research focusing on the N_2_ reduction reaction via electrocatalysis using carbon‐based materials as supports for metal SAs.^[^ ^270^, ^271^ ^]^ The first example in photocatalysis was demonstrated by Xie and co‐workers.^[^ ^272^ ^]^ They decorated Cu SAs on a g‐C_3_N_4_ catalyst and achieved an NH_3_ yield of 186 µmol g^−1^ h^−1^ under visible light irradiation, with a quantum efficiency of 1.01% under 420 nm monochromatic light (**Figure** **15**a–c). The coordination of Cu SAs in g‐C_3_N_4_ contributes to a large amount of active isolated π‐electrons and superior adsorption ability on the positively charged Cu ions, resulting in enhanced N_2_ activation ability and superior photocatalytic N_2_ reduction performance. (It should be noted that the results were based solely on in situ FTIR analysis and were not confirmed using isotope labeling.)

In the meantime, numerous studies have reported on SAs for photocatalytic NH_3_ production, with Ru SAs being the most frequently used, including those supported on TiO_2_,^[^ ^273^, ^274^, ^275^ ^]^ C_3_N_4_,^[^ ^215^ ^]^ Fe_2_O_3_,^[^ ^276^ ^]^ CoO,^[^ ^277^ ^]^ H*_x_* MoO_3−_ *_y_*.^[^ ^189^ ^]^ Generally, a strong metal–support interaction is reported to facilitate the adsorption and activation of N_2_ molecules. These interactions stabilize the N_2_ molecules on the catalyst surface, reducing the energy barrier for the NRR and enabling NH_3_ production under milder conditions compared to conventional methods. For example, Ru SAs decorated TiO_2_ nanosheets have been reported to improve the NH_3_ production rate in comparison to bare TiO_2_ nanosheets.^[^ ^274^ ^]^ The isolated Ru atoms, positioned at oxygen vacancies on the TiO_2_ surface, were found to suppress the hydrogen evolution reaction, thereby facilitating N_2_ chemisorption and improving charge carrier separation, which ultimately leads to enhanced N_2_ photoreduction.

Apart from Ru SAs, other metal SAs have also been reported to enhance the photocatalytic N_2_ reduction reactions, for example, Fe SAs,^[^ ^278^, ^279^, ^280^ ^]^ Cu SAs,^[^ ^272^, ^281^ ^]^ Pd SAs,^[^ ^282^ ^]^ and La SAs,^[^ ^283^ ^]^ etc. Some studies focus primarily on theoretical insights, such as the case of single Fe atoms anchored on TiO_2_ (001),^[^ ^284^ ^]^ which were calculated to exhibit good activity for NRR due to an energetically favorable pathway for the rate‐determining step (N_2_* + H* → NNH), the slightly oxidized Fe was also proposed to act as an active site for N_2_ activation and reduction.

Mo SAs have also been reported to enhance photocatalytic N_2_ fixation performance on C_3_N_4_,^[^ ^285^ ^]^ as the low‐coordinated Mo centers serve as active sites for N_2_ chemisorption and activation, leading to high photocatalytic activity for NH_3_ production.

Defect engineering of the support material, as previously discussed, has been widely used to enhance photocatalytic N_2_ fixation performance by improving charge transfer. In the case of SAs, defects can also provide anchoring sites, increasing stability and preventing aggregation during the reaction. For example, TiO_2_ with oxygen vacancies have been employed to anchor Ru SAs and promote N_2_ fixation performance.^[^ ^273^ ^]^

Apart from the mostly investigated TiO_2_ and C_3_N_4_ systems, organic semiconductors such as MOFs,^[^ ^286^, ^287^ ^]^ and COFs^[^ ^288^, ^289^ ^]^ have also been used for photocatalytic N_2_ fixation. These organic light absorbers have predesignable chemical structures and highly ordered crystalline nature, which could provide ideal coordination environments for SAs and prevent their aggregation, and allow precise tuning of atomic properties to improve catalytic performance.^[^ ^290^ ^]^ For example, He et al. prepared a series of Au‐SAs anchored porphyrin‐based COF photocatalyst for photocatalytic N_2_ fixation (Figure 15d–f),^[^ ^289^ ^]^ and by introducing electron withdrawing groups within the COF structure, the NH_3_ production rates can be optimized and reach a maximum of 427.9 ± 18.7 µmol g^−1^ h^−1^ (Figure 15g).

Despite significant efforts, the yield of NH_3_ in photocatalytic N_2_ fixation remains far below the levels required for practical industrial applications. The challenges and pitfalls in N_2_ reduction, i.e., even the reliable detection of reaction products (positive results often due to contamination, lack of isotope labeling experiment) are even more severe than those encountered in CO_2_ reduction.^[^ ^291^, ^292^ ^]^ This problem is particularly acute in organic semiconductor system such as C_3_N_4_, due to the presence of nitrogen‐containing functional groups in the catalyst structure.

The common issue with providing reliable date is well described in literature,^[^ ^293^ ^]^ and a standardized and rigorous protocol has been proposed—but is rarely used in practice.

### 6.4 Photocatalytic Pollutant Removal

Photocatalysis has proven to be an effective method for degrading and removal of various pollutants, including air pollutants like NO*_x_* and volatile organic chemicals and wastewater contaminants such as heavy metal ions, organic dyes, and antibiotics, as extensively studied in the works of Fujishima and co‐workers and summarized in many reviews.^[^ ^37^, ^294^, ^295^ ^]^ Such removal processes can either be based on intermediate formation of reactive oxygen species (ROS) by either a conduction band mechanism (reduction of environmental O_2_) to ROS, or by a valence band mechanism (oxidation of water to ROS by hole capture, or the direct oxidation of organic molecules by hole capture). Which of the mechanisms dominate, depends strongly on the energetics of the semiconductor and the surface adsorption characteristic of the pollutant. A low valence band position of the semiconductor typically favors the generation of hydroxyl radicals (•OH) as the dominant pathway due to its strong oxidative potential. A higher conduction band position can facilitate the reduction of cationic pollutants, such as heavy metal ions, via electron transfer. Additionally, the nature of the pollutant plays a crucial role: anionic pollutants tend to be more effectively degraded through oxidation mechanisms (hole capture), while cationic pollutants are easier to be reduced (electron capture). If ROS are the main reaction pathway, typically the critical reaction step is reaction with pollutants, converting them into less harmful by‐products such as carbon dioxide and water. Moreover, also photo‐Fenton system (H_2_O_2_, peroxymonosulfate (PMS), and peroxydisulfate) and coupled photo/H_2_O_2_ (O_3_ or PMS) systems have been explored.^[^ ^296^ ^]^ SACs have also shown some promise in this area. Among the many semiconductors used in photocatalytic pollutant removal, TiO_2_ and C_3_N_4_ have been the most extensively studied. Some examples include Pt SAs on TiO_2_ for degrading formaldehyde and toluene,^[^ ^297^, ^298^ ^]^ Pd SAs on C_3_N_4_ for NO removal,^[^ ^187^ ^]^ and Ag SAs and Fe SAs on C_3_N_4_ for antibiotic degradation.^[^ ^299^, ^300^ ^]^

**Table** **4** summarizes some single‐atom photocatalysts used for photocatalytic pollutant removal.

**Table 4:** Summary of SACs for photocatalytic pollutant removal.

SAs	Substrate	Pollutant	Reaction phase	Reaction way	Refs.
Cu	ZnO/GPET	Methylene blue	Liquid	Direct photocatalysis	[303]
Fe	C_3_N_4_	Sulfamethoxazole	Liquid	Photocatalysis/PMS	[300]
Fe	C_3_N_4_	Ciprofloxacin	Liquid	Photo‐Fenton reaction	[302]
Ni	C_3_N_4_	Gemfibrozil	Liquid	Direct photocatalysis	[304]
Au	CQDs/C_3_N_4_	Naproxen	Liquid	Direct photocatalysis	[305]
Ag	C_3_N_4_	Naproxen	Liquid	Direct photocatalysis	[299]
Ag	C_3_N_4_	Tetracycline	Liquid	Direct photocatalysis	[306]
In	C_3_N_4_	Tetracycline, ciprofloxacin	Liquid	Direct photocatalysis	[301]
La, Er	TiO_2_	Gaseous O‐xylene	Gas	Direct photocatalysis	[307]
Pt	C_3_N_4_	Sulfonylurea herbicide	Liquid	Direct photocatalysis	[308]
Mn	N‐doped GO	Sulfanilamide	Liquid	Direct photocatalysis	[309]
Mg	C_3_N_4_	NO	Gas	Direct photocatalysis	[310]
Pd	C_3_N_4_	NO	Gas	Direct photocatalysis	[187]
Pd	TiO_2_	NO	Gas	Direct photocatalysis	[311]
Mn	TiO_2_	Formaldehyde	Gas	Direct photocatalysis	[312]
Pt	TiO_2_	Formaldehyde	Gas	Direct photocatalysis	[297]
Pt	TiO_2_	Toluene	Gas	Direct photocatalysis	[298]

Zhang et al.^[^ ^301^ ^]^ prepared C_3_N_4_ with nitrogen vacancies to coordinate In SAs and achieved a degradation rate of 0.101 min^−1^ for tetracycline, a 50‐fold increase compared to pristine C_3_N_4_. They claimed that the isolated In atoms, combined with neighboring N2C‐type nitrogen vacancies, create defect states that act as electron traps, accelerating the accumulation of long‐lived holes at the In atom sites by trapping photogenerated electrons, leading to the excellent degradation performance. Zhao et al.^[^ ^300^ ^]^ prepared a Fe SAs decorated C_3_N_4_ catalyst with a Fe–N_4_ coordination structure for the degradation of sulfamethoxazole (SMX) using PMS for activation. They achieved a 98.7% SMX degradation within 6 min, with a TOF of 5.27 min^−1^, which is 2–6 times higher than that of conventional Fe‐containing catalysts. The study identified Fe SAs in the catalyst as the primary active sites for PMS activation under visible light irradiation, i.e., photogenerated electrons accelerated the Fe(II)/Fe(III) cycling, while photogenerated holes contributed to the generation of singlet oxygen (^1^O_2_) thus promoting the degradation of SMX. Su et al.^[^ ^302^ ^]^ reported a photo‐Fenton system for ciprofloxacin degradation using Fe SAs decorated C_3_N_4_. The Fe SA decorated C_3_N_4_ exhibited an 18‐fold enhancement in ciprofloxacin degradation compared to pristine C_3_N_4_. This significant improvement is attributed to the increased local electron density on the Fe single sites, leading to high catalytic efficiency in H_2_O_2_ activation and substantial generation of hydroxyl radicals (•OH) for the removal of organic pollutants.

### 6.5 Photocatalytic Organic Synthesis

Photocatalytic organic synthesis is often considered as one of the most sustainable and economical strategies for the production of high‐value organic compounds. Photogenerated electrons, holes, and various radical species act as reducing or oxidizing agents in organic transformation reactions. The field is still far from being well explored, mainly because of the nonspecific formation of reaction products. If one considers previous discussion on photocatalytic reactions for organic pollutant removal—via direct red‐ox reactions or via an intermediate ROS species—it is clear that achieving selectivity in these types of reactions is a major issue.

In other words, any measure that sharpens reaction selectivity may constitute a significant step to applicability. Therefore, SAs are being increasingly investigated in this field. So far, single‐atom photocatalysts have been used in different photocatalytic reactions, such as oxidation and reduction reactions, coupling reactions (e.g., C–C and C–O coupling), hydrogenation, sulfonation, etc.^[^ ^313^ ^]^ **Table** **5** summarizes efforts using SACs for photocatalytic organic synthesis.

**Table 5:** Summary of SACs for photocatalytic organic synthesis.

Catalysts	Photocatalytic reaction	Products	Performance	Refs.
Ni SAs/TiO_2_	Sulfonation of enamides	Amidosulfones	Yield: 99%	[316]
Cu SAs/TiO_2_	Photosynthesis of urea	Urea	Production rate: 7.2 µmol h^−1^ g^−1^	[315]
Pd SAs/CdS	Ethanol reforming	1,1‐Diethoxyethane and H_2_	Selectivity: 100% Production rate: 35.1 mmol h^−1^ g^−1^	[188]
Pt SAs/TiO_2_	Acetone reforming	2,5‐Hexanedione and H_2_	Selectivity: 93% Production rate: 3.87 mmol h^−1^ g^−1^	[317]
Ni SAs/C_3_N_4_	Coupling of 4′‐bromoacetophenone with methanol	4′‐Methoxyacetophenone	Yield: 97%	[318]
Zn‐HOF	Benzylamine oxidative coupling	Benzylidenebenzylamine and H_2_	Conversion: 100%, selectivity: 91%	[319]
Fe SAs/C_3_N_4_	Oxidation reaction of ethylbenzene	Acetophenone	Conversion: 99%, selectivity: 99%	[320]
Ni SAs/C_3_N_4_	C─O coupling reaction	4′‐Methoxyacetophenone	Production rate: 4.8 mmol h^−1^ g^−1^	[314]
Pt SAs/COF	Benzylamine oxidative coupling	Benzylidenebenzylamine and H_2_	Production rate: 477.3 µmol h^−1^ g^−1^ Selectivity: 98%	[321]
Cu SAs/C_3_N_4_	Suzuki cross‐coupling reaction	4‐Phenyltoluene	Yield: 98%	[322]

An example is the work of Bajada et al.^[^ ^314^ ^]^ who developed a C_3_N_4_‐supported Ni SAs photocatalyst for the C–O coupling of carboxylic acids and alkyl halides. They found that light illumination significantly enhances the reaction. Specifically, the production rate increased from 0.6 mmol h^−1^ g^−1^ without illumination to 4.2 mmol h^−1^ g^−1^ with illumination. They propose that light triggers an energy transfer process, leading to the metallophotocatalytic production of the ester.

Li et al.^[^ ^315^ ^]^ decorated TiO_2_ with Cu SAs and used it for the photosynthesis of urea from N_2_, CO_2_, and H_2_O, achieving a production rate of 7.2 µmol h^−1^ g^−1^. Using quasi‐in situ characterizations, they revealed the reaction dynamics, showing that the reversibility of single‐atom copper and its rapid extraction rate of photogenerated electrons are beneficial for molecular activation and C–N coupling, thus promoting urea photosynthesis.

Nevertheless, these few examples illustrate that it still represents a largely unexplored field.

### 6.6 Other Applications

Single‐atom cocatalysts have also shown promising potential in various photocatalytic applications, including H_2_O_2_ production, etc.^[^ ^323^ ^]^ Zhang et al.^[^ ^324^ ^]^ reported a high‐loading Ni single‐atom photocatalyst for efficient H_2_O_2_ synthesis in pure water, achieving an apparent quantum yield of 10.9% at 420 nm and a solar‐to‐chemical conversion efficiency of 0.82%. The results indicate that the evolution of the active site structure reduces the formation energy barrier of *OOH and suppresses O═O bond dissociation, thereby improving H_2_O_2_ production activity and selectivity.

Many other applications are conceivable—in principle all that are based on photocatalytic effects (self‐cleaning, wetting, drug delivery, antibacteria, etc.)—and may be influenced by suitable cocatalysts. Most remarkable may be unexpected catalytic effects of SAs, such as the recently shown catalytic feature of SAs on TiO_2_ for the OER on photoanodes, where Pt SAs significantly enhance OER performance, whereas NPs show no such effect.^[^ ^14^ ^]^

## 7 Some Considerations and Future Perspectives

The field of photocatalysis has been profoundly impacted by the advent of Pt SA cocatalysts, marking a significant leap forward in catalytic efficiency and sustainability. For photocatalysis, as well as for classic “chemical catalysis” transformative potential lies I) in the high degree of noble metal utilization increase, but even more so in II) the SAs unique catalytic properties.

### 7.1 Save Material, Save Money

The first aspect I) allows to use high efficiency (high TOF materials) such as Pt in spite of its cost in photocatalytic applications such as hydrogen production, CO_2_ reduction, and pollution remediation in a highly economic and possibly even sustainable way. As long as scaling laws hold (one SA is replacing one cocatalytic nanoparticle), the gain in Pt usage is a factor of 100–5000. Currently, the worldwide known Pt reserves are expected to satisfy the global needs for another 50–100 years,^[^ ^325^, ^326^ ^]^ so an according gain of a factor 1000 may be remarkable. Please note, however, a few facts: the main use of Pt today (≈50%) is in automotive catalysts, so to use SAs in that application would be most impactful; recycling of Pt is currently only 25% of the Pt use (i.e., this is another impactful factor for establishing a sustainable Pt use). Note also that a shift to electro‐based cars would accordingly double the availability of Pt. Less scientific may be that almost 90% of the world‐wide Platinum Group MetalsPGM reserves are located in South Africa. So, better get friends with it!

Nevertheless, as outlined before, particularly in photocatalysis that relies mostly on cheap abundant semiconductive materials, turning the cocatalyst Pt into a negligible cost factor in view of an entire photocatalytic system is monetarily highly beneficial.

For example, under standard photocatalytic H_2_ production conditions using AM 1.5G as the light source, roughly a coverage of just one Pt atom per 10 nm^2^ is sufficient to handle all incoming electrons and achieving maximum H_2_ production efficiency. This corresponds to a Pt cost of ≈0.1–0.2 cents per m^2^. From an economic perspective, if the photocatalyst demonstrates long‐term stability, this approach becomes highly cost‐effective.

Due to these economic considerations, most current research focuses on directly replacing nanoparticle catalysts with single‐atom counterparts.

### 7.2 Further Optimization of Use of SA in Photocatalyst Systems

Except for just replacing Pt nanoparticles, where in traditional many‐atom‐nanoparticles, the TOF is limited by factors such as poor atom accessibility, suboptimal positioning, and slower electron transfer, in view of further optimization the strategy is “making the most of SAs,” by: a) deposit the minimum SA amount that is needed, not more, b) deposit SAs in a most reactive configuration, and c) deposit SAs only at locations where they are needed.

a) Interplay of semiconductor and SA active site

A key point in minimizing the loading of Pt SAs (while maintaining a maximum performance) is designing structures with light harvesters (electron collectors) that can optimally exploit the very high maximum turnover frequency of Pt SAs. Under maximized TOF conditions each atom is operating at its full potential. In the interplay of SA and light‐harvesting, the substrate should be optimized as well in view of light absorption, charge carrier mobility, and charge transfer from the substrate to the SA and onward to the reaction, so that each SA can be fed with a nearly saturating flux of electrons and thus can operate at a maximum TOF.

In other words, well‐defined semiconductors that have “good” electronic properties (high carrier transport length, surface harvesting length) can drastically decrease the need for a high Pt SA loading density. In contrast, using highly defective semiconductors (or even semi‐isolators such as many MOFs, zeolites, etc.) leads to the need of a high cocatalyst loading and/or underperforming systems.

b) Deposition in most active surface configuration

To reach a maximized activity of SAs, they should preferentially be placed at “hot spots.” Different deposition methods may lead to different surface configurations of SAs on surfaces. Physical deposition techniques have a tendency to lead to statistical SA distribution on surfaces. Such surfaces may be activated (thermally treated) to let SAs surface‐diffuse to stable and/or most active positions. Techniques that lead to SA‐anchoring in a highly active surface position are some approaches based on reactive deposition of SAs—they can show self‐homing characteristics. Self‐homing refers to the process by which single atoms naturally migrate and anchor themselves at the most reactive sites on a substrate during deposition. This phenomenon occurs without the need for external intervention, as the atoms “self‐select” the optimal locations where they can deliver maximum catalytic efficiency. These locations are typically characterized by high surface energy, defects, or specific electronic coordination configurations that trap the atom and at the same time favor catalysis. For instance, in photocatalytic water splitting, Pt atoms can anchor themselves at most reactive sites on semiconductor surfaces, such as TiO_2_ or C_3_N_4_, enable highly efficient electron transfer, leading to significantly enhanced hydrogen evolution rates. This targeted positioning of Pt atoms can achieve catalytic performance with only a fraction of the platinum typically required in conventional nanoparticle systems, or in comparison with randomly loaded or excess loaded Pt SA structures.

c) Place SAs only on electron exit sites

Further increase in Pt utilization efficiency can be reached, if SAs are only deposited on semiconductive structures at locations where they are truly effective. Many light absorber structures provide defined electron and hole exit sites (e.g., internal junctions, molecular electron sinks). Accordingly, electron transfer catalysts (SAs) are only needed at these surface sites—deposits on the rest of the surface are either wasted or may even be counterproductive.

An example are preferentially faceted single‐crystalline sheets, which can enhance photocatalytic activity due to internal nanojunctions leading to charge separation where electrons migrate and exit at the (101) facets while holes accumulate and exit at the (001) facets. Consequently, a cocatalyst for H_2_ evolution is only required on the (101) facets. Such structures have the potential to provide a very high SA utilization efficiency.

### 7.3 Unique Features of SAs

A most remarkable feature, maybe THE most remarkable feature of SAs is their potential to alter and unlock unusual catalytic mechanisms and reaction pathways. For example, in photocatalytic CO_2_ reduction, Pt SAs significantly enhance CH_4_ production, whereas Pt NPs primarily drive the competing water‐splitting reaction to produce H_2_.^[^ ^17^ ^]^ Even more, in photoelectrochemical OER, Pt SAs show (against classic expectations) a remarkable cocatalytic effect, while Pt NPs show neglectable impact.^[^ ^14^ ^]^

Despite significant success in revealing new reaction pathways, the potential to alter and unlock unusual catalytic mechanisms and reaction pathways remains largely untapped. It is certainly a research field where most exciting and radically new findings are possible (likely again a careful design of SA catalyst anchoring and configuration).

### 7.4 Some Remarks to the Current state of Research

#### 7.4.1 Charge Carrier Mobility

A critical observation in current research on the design of photocatalysts is the lack of attention regarding the use of optimized light harvester systems in conjunction with an SA cocatalyst. Effective photocatalysis relies not just on the catalytic material but also on how well the semiconductor absorbs, converts, and utilizes light. Unfortunately, many studies overlook the need for carefully engineered systems that combine efficient light absorption with optimal charge carrier transfer to SA sites. Metal–organic compounds, which are often used in SA catalysis, tend to suffer from excessive SA catalyst loading—the same holds for the use of poor semiconductors that are either poor conductors or even insulators (e.g., ZrO_2_) with low electron mobility. As a result, these systems require a high SA loading to achieve meaningful catalytic activity, which undermines the efficiency‐gains that single‐atom catalysts are meant to deliver. This mismatch between SA catalysts and poorly performing semiconductors not only increases material costs but also fails to optimize the photocatalytic performance potential. Or in other words, for low‐quality‐semiconductors that intrinsically need a high loading of a cocatalyst, Pt must be replaced by a “cheap” cocatalyst—giving up on the exceptionally high performance of Pt type materials.

#### 7.4.2 Stability

One of their greatest challenges in the SA field remains improving the stability of SACs (without impairing their reactivity). Main challenges are not only i) the “thermally activated” agglomeration of SAs into clusters or nanoclusters during treatments or extended use in photo(electro)catalytic reactions, but also ii) the mobilization of anchored single atoms by reaction intermediates, and iii) the strong binding of reaction intermediates to SAs (poisoning). While improving the metal–support interaction or increasingly chemically or sterically trapping can help prevent SA agglomeration, it often goes along with alteration (degradation) of the reactivity. Here certainly novel concepts are needed.

#### 7.4.3 Methods

In the review we discuss common characterization techniques that are crucial for the characterization of SAs on substrates and we highlighted some points of caution when using them. One may deduce that there is a need for monitoring the behavior of SAs in real time under reaction conditions. Methods such as in situ IR, XPS, and in situ XAS and electron microscopy enable researchers to capture the dynamic interactions of SAs, revealing changes in oxidation states, coordination environments, and reaction intermediates during photocatalysis. Nevertheless, these techniques should also be used cautiously, many in situ tools like in situ TEM or XAS may strongly modify the photo(electro)chemical behavior of the target sample. Therefore, careful consideration should be given to avoid drawing inappropriate conclusions based on changes induced by the measurement itself. To improve the reliability and accuracy of investigations, developing well‐defined model systems, experimental setups, and carrying out proper control experiments, is essential.

#### 7.4.4 Interplay of Experiment and Theory

Although SACs have shown remarkable promise in various catalytic applications, the exact mechanisms by which single atoms facilitate photocatalytic reactions are often not fully understood. A comprehensive mechanistic understanding is desired for rational catalyst design, as it would provide a more straightforward optimization of the catalytic process, and its efficiency and selectivity. This deepening of understanding not only requires advanced characterization techniques for SAs, but also needs theoretical investigations as a support to better understand the reaction dynamics.

One key challenge is identifying the active sites and reaction intermediates involved in SAC‐driven photocatalysis. Due to the isolated nature of the single atoms, their interaction with the support, light, and reactants can be highly complex and context‐dependent.

Recent advancements in theoretical studies, particularly employing DFT in conjunction with carefully designed experiments, have begun to illuminate the reaction pathways and energy barriers associated with SAs. For example, band structure calculations and calculations considering excited states help to elucidate the absorption properties of photocatalysts, providing insights into how SA integration can extend or enhance light absorption. Adsorption energy calculations reveal the most favourable binding sites and the thermodynamics of key intermediates, shedding light on the reaction pathways and identifying potential rate‐limiting steps. Additionally, charge transfer analyses illustrate how single atoms influence the distribution and mobility of charge carriers, which is critical for optimizing photocatalytic efficiency. These theoretical insights, when validated through experimental studies, provide crucial guidance for ultimately advancing the field of photocatalysis. However, it is important to note that theoretical models often rely on simplified assumptions that may not fully capture the complexity of real catalytic systems. Factors such as solvent effects, temperature variations, and support interactions can be difficult to accurately model and may lead to discrepancies between theoretical predictions and experimental outcomes. Thus, careful calibration and validation of theoretical results with experimental data are essential to ensure their reliability and applicability to practical systems.

In spite of these calls for caution, SAs are most ideal platform for a fruitful overlap of theory and experiments, and a truly close interplay can be a main propellant of the field.

#### 7.4.5 Toward Technology

Scaling up the production of SACs is not yet an urgent demand but will become essential for future development. This requires not only scaling up the synthesis of SAs but also the production of large‐scale semiconductor supports. Different methods may be needed depending on the catalyst format. For instance, wet‐chemistry techniques are suitable for both particulate and film catalysts, while physical or chemical deposition techniques typically are used in connection with thin layers. Ensuring precise and reproducible manufacturing processes is crucial, with a focus on parameters like the dispersion, uniformity, and loading amount of SAs in large‐scale synthesis. Moreover, the design of large‐scale photoreactors is still in its infancy.

Integrating these catalysts into existing or future industrial processes demands innovative reactor designs and modifications to accommodate their high reactivity and sensitivity to environmental conditions. Addressing these challenges will require considerable engineering efforts.

Nevertheless, old wisdom tells: When money is to be made, technology often is ahead of science.

#### 7.4.6 Nomenclature

In literature, often clear distinctions and definitions between different approaches to SA‐based work are established, proposed, or demanded (e.g., SA photocatalyst vs organometallic, homogeneous vs heterogeneous approaches, supported single‐atom catalysts vs molecular catalysts, oxide‐supported vs carbon‐supported systems, doping vs adsorbing, etc.)—this although in many cases and from a fundamental mechanistic viewpoint, processes are identical and the border of disciplines is very fluent.

From a pragmatic viewpoint, one may argue that one does not need to care that much if a Pt defines itself as oxygen‐, nitrogen‐coordinated, inorganic, organic surface complexed or coordinated, tethered or freely dynamic, if it does its job (and saves the Pt‐world), all is very fine.

#### 7.4.7 Final, Future and Fiction

In summary, while SA cocatalysts present transformative potential in catalysis and photocatalysis, fully unlocking their capabilities requires a deeper understanding of the underlying mechanisms and concepts. The integration of advanced characterization techniques, theoretical modeling, and innovative experimental designs is proving pivotal in controlling the placement, stability, and functionality of SAs, and to drive further the progress in the field.

Many issues discussed in this review may be common knowledge in the field. However, there are many papers published that seem to have one view‐angle perspective—we believe this is due to the overlap of classic fields that approach SA photocatalytic work from classic catalyst‐knowledge, from semiconductor physics, electrochemistry, coordination‐chemistry, or coming from surfaces and interfaces background. Therefore, we apologize if the review addresses many fundamental points, but we aim to consolidate foundation points, key concepts and factors, where in our opinion clarification is warranted.

The review moreover discusses future directions research and we feel that the full potential of SAs may still be lying ahead.

Examples of work that need further dedication, is namely the exploration of the full extent of the width of variations of reactivity that can be achieved with an SA. Can we turn Fe into Au, or Cu to Pt? This by an ideal selection of size, coordination, and substrate interactions? Many supports still offer unexplored features of electronically, sterically, and chemically tuneable functionality.

Like the search for the philosopher's stone is the search for the “superactive SA”—it is related to the open question: what is really the maximum TOF of a Pt SA—and how can we maximally tweak it.

Another widely open field is the placement of multiple different SAs that may interact synergistically either in a common reactive site or in surface‐spaced arrangement, fulfilling individual tasks in a catalytic process. Here a large degree of further innovation is needed in placement design and methods of placement to fully assess the potential of such multi‐SA catalysts.

Nevertheless, in view of future, and in spite of a plethora of well‐thought‐out strategies, the most important feature of the future, as always in research, is “the unexpected.”

The unexpected is the seed of the breakthrough! And this, in our view, is particularly true for “single‐atom handling.”

## Conflict of Interest

The authors declare no conflict of interest.
